# Systematic analysis of specific and nonspecific auxin effects on endocytosis and trafficking

**DOI:** 10.1093/plphys/kiab134

**Published:** 2021-03-18

**Authors:** Madhumitha Narasimhan, Michelle Gallei, Shutang Tan, Alexander Johnson, Inge Verstraeten, Lanxin Li, Lesia Rodriguez, Huibin Han, Ellie Himschoot, Ren Wang, Steffen Vanneste, Judit Sánchez-Simarro, Fernando Aniento, Maciek Adamowski, Jiří Friml

**Affiliations:** 1 Institute of Science and Technology (IST), Klosterneuburg 3400, Austria; 2 Department of Plant Biotechnology and Bioinformatics, Ghent University, Gent, Belgium; 3 VIB Center for Plant Systems Biology, Ghent, Belgium; 4 Departamento de Bioquímica y Biología Molecular, Facultad de Farmacia, Universitat de Valencia, 46100 Burjassot, Spain

## Abstract

The phytohormone auxin and its directional transport through tissues are intensively studied. However, a mechanistic understanding of auxin-mediated feedback on endocytosis and polar distribution of PIN auxin transporters remains limited due to contradictory observations and interpretations. Here, we used state-of-the-art methods to reexamine the auxin effects on PIN endocytic trafficking. We used high auxin concentrations or longer treatments versus lower concentrations and shorter treatments of natural indole-3-acetic acid (IAA) and synthetic naphthalene acetic acid (NAA) auxins to distinguish between specific and nonspecific effects. Longer treatments of both auxins interfere with Brefeldin A-mediated intracellular PIN2 accumulation and also with general aggregation of endomembrane compartments. NAA treatment decreased the internalization of the endocytic tracer dye, FM4-64; however, NAA treatment also affected the number, distribution, and compartment identity of the early endosome/trans-Golgi network, rendering the FM4-64 endocytic assays at high NAA concentrations unreliable. To circumvent these nonspecific effects of NAA and IAA affecting the endomembrane system, we opted for alternative approaches visualizing the endocytic events directly at the plasma membrane (PM). Using total internal reflection fluorescence microscopy, we saw no significant effects of IAA or NAA treatments on the incidence and dynamics of clathrin foci, implying that these treatments do not affect the overall endocytosis rate. However, both NAA and IAA at low concentrations rapidly and specifically promoted endocytosis of photo-converted PIN2 from the PM. These analyses identify a specific effect of NAA and IAA on PIN2 endocytosis, thus, contributing to its polarity maintenance and furthermore illustrate that high auxin levels have nonspecific effects on trafficking and endomembrane compartments.

## Teaser

Natural and synthetic auxins affect various aspects of the endomembrane system at high concentrations, but promote clathrin-mediated endocytosis of the PIN2 auxin transporter at low concentrations.

## Introduction

A multitude of developmental processes throughout the lifecycle of a plant, such as organ formation, embryonic axis establishment, and tropic responses, are regulated by the local accumulation and asymmetric distribution of the growth regulating hormone auxin (Bargmann et al., 2013; Lavy and Estelle, 2016; Gallei et al., 2020). The formation and maintenance of directional polar auxin transport (PAT) between cells is regulated by polar-localized, plasma membrane (PM)-based auxin efflux carries from the PIN protein family (Adamowski and Friml, 2015; Grones and Friml, 2015).

Auxin itself has the ability to dynamically change the subcellular localization of PINs at the PM by forming a feedback loop between auxin signaling and transport (Ravichandran et al., 2020). This was proposed to facilitate the rise of new polarized routes of auxin transport (Mazur et al., 2016; Prát et al., 2018; Mazur et al., 2020a). This canalization mechanism plays a role in the developmental processes involving flexible formation of new vasculature, such as leaf venation (Scarpella et al., 2006), vasculature regeneration after wounding (Sauer et al., 2006; Mazur et al., 2016; Mazur et al., 2020b), and connecting organs at the shoot apical meristem (Benkova et al., 2003) or lateral buds to pre-existing vasculature (Balla et al., 2011; Shinohara et al., 2013; Zhang et al., 2020). Furthermore, auxin-mediated changes in PIN polarity were also observed during apical-basal axis formation during embryogenesis (Robert et al., 2018, 2013; Wabnik et al., 2013) or during termination of shoot bending responses (Rakusová et al., 2016, 2019).

Dynamic changes in PIN polarity, including those mediated by auxin itself, have been linked to constitutive PIN cycling from and to the PM (Geldner and Palme, 2001; Dhonukshe et al., 2007; Kleine-Vehn, 2008a, 2008b). Constitutive internalization and recycling of PINs are also important for the regulation of steady-state PIN polarity (Kleine-Vehn et al., 2011; Glanc et al., 2018), and interference with this process, for example, by disturbing phosphorylation and dephosphorylation switches, leads to severe growth defects (Barbosa et al., 2018; Grones et al., 2018).

The constitutive internalization of PINs was shown to be through clathrin-mediated endocytosis (CME; Dhonukshe et al., 2007; Kitakura et al., 2011; Adamowski et al., 2018). By nonspecifically interfering with CME, auxin could increase its own efflux by stabilizing PINs at the PM and consequently organize PAT within tissues (Paciorek et al., 2005; Robert et al., 2010). This is unlike most other hormones, which regulate the endocytosis of their own receptors and related proteins but not overall endocytosis of the cell (Irani et al., 2012; Di Rubbo et al., 2013; Belda-Palazon et al., 2016). However, this is complicated by the fact that auxin imparts two opposite effects on PINs. Studies have shown that over the long term (>2 h), auxin causes the loss of PIN2 and its subsequent degradation (Abas et al., 2006). Notably, canalization-related PIN polarity changes at the PM occur within a similar time-frame (Vieten et al., 2005; Sauer et al. ,2006; Baster et al., 2013). In contrast, short-term auxin treatments (<1 h) stabilize PINs at the PM by inhibiting CME (Paciorek et al., 2005; Robert et al., 2010; Oochi et al., 2019).

Various auxin effects on PIN trafficking have been observed using different auxin isoforms; however, the synthetic auxin 1-naphthalene acetic acid (NAA) was used preferentially in most studies (Paciorek et al., 2005; Abas et al., 2006; Robert et al., 2010) due to its reported higher stability in comparison to the natural auxin, indole-3-acetic acid (IAA; Paciorek et al., 2005). However, there have been a number of recent reports that show that the natural auxin, IAA, mediates its effect rapidly (faster than a minute) and remains active and effective for an extended period of time, such as for 48 h (Eliasson et al., 1989; Fendrych et al., 2018). The half-life of active IAA was estimated by UPLC-MS (ultra performance liquid chromatography – mass spectroscopy) to be 35 h (Paponov et al., 2019b). The property of auxin to interfere with endocytosis and related processes was mainly inferred indirectly by the use of Brefeldin-A (BFA). BFA is a fungal toxin that inhibits trafficking from endosomes to the PM by targeting the guanosine nucleotide exchange factor of adenosine-ribosylation-factor type small GTPases (ARF GEF), known as GNOM (Geldner et al., 2003; Naramoto et al., 2014). Consequently BFA treatment causes reversible aggregation of endosomes into “BFA bodies” in Arabidopsis roots (Paciorek et al., 2005; Kania et al., 2018; Zhang et al., 2020). As BFA has no direct effect on the endocytic rate of cargoes from the PM to the endosomes (Naramoto et al., 2010), observation of endocytosed cargoes in the BFA bodies has been extensively used as an indirect measure of the internalization rate (Geldner and Palme, 2001). When co-treating with BFA and auxin, intracellular accumulation of PIN1, PIN2, and other cargo proteins in BFA bodies is inhibited, suggesting a decrease in the endocytosis of these cargoes (Paciorek et al., 2005). The auxin effect on endocytosis, more specifically on CME, has been further supported by auxin-mediated inhibition of internalization of the endocytic tracer FM4-64 (Jelínková et al., 2010) and reduced clathrin incidence at the PM (Paciorek et al., 2005; Robert et al., 2010). This auxin effect on endocytosis is non-transcriptional and does not require the canonical TIR1/AFB auxin pathway, but has been linked to the Auxin Binding Protein 1 (ABP1) based on the gain-of-function and generic, conditional loss-of-function studies (Robert et al., 2010; Grones et al., 2015). However, due to the lack of obvious phenotypic defects in the verified *abp1* knockout alleles (Gao et al., 2015; Grones et al., 2015; Michalko et al., 2016; Gelová et al., 2020), the cellular function of ABP1 remain to be understood.

In addition to the short-term auxin inhibitory effect on overall endocytic processes, prolonged auxin treatments lead to a decrease in the PM or microsomal fraction incidence of some cargoes, in particular, PIN2. This would imply an increase in endocytosis and degradation (Abas et al., 2006; Baster et al., 2013). The use of the photo-convertible fluorescent variants of PIN2 confirmed this auxin-mediated PIN2 degradation and also revealed a significant contribution of de novo synthesized PIN2 to the PIN2 protein pool accumulating in BFA bodies (Jasik and Elmon, 2016; Salanenka et al., 2018). Moreover, PIN1 accumulation in the BFA bodies can be inhibited not only by active auxins, such as NAA, but also by its inactive analog 2-NAA (Paponov et al., 2019b); however, it was previously shown that 2-NAA is less effective in mediating the process (Paciorek et al., 2005).

All these partly contradictory observations and interpretations are hard to reconcile into a coherent mechanistic understanding of auxin effects on endocytic trafficking. Thus, given the potential importance of these processes and their auxin regulations for feedback control of auxin homeostasis (Paciorek et al., 2005) or coordinated polarization and auxin channel formation (Wabnik et al., 2010; Mazur et al., 2020a), it is paramount to revisit these questions using state-of-the-art visualization and genetic tools.

Here we clarify the previously observed auxin effects and their interpretations, and further present new insights into auxin regulation of endocytosis and constitutive endocytic trafficking. We used the synthetic auxin NAA and the natural auxin, IAA, for all our studies. To differentiate between specific and nonspecific auxin effects, we chose incremental concentrations from 10 nM to 100 µM and treatment durations of 5 min–2 h. We further provide: (1) a better characterization of the effects of NAA and IAA on different cellular processes including CME and intracellular trafficking; (2) comparisons of the effects between NAA and IAA on different cargoes; and (3) the identification of a rapid, specific endocytic auxin effect, during which NAA and IAA, even at very low concentrations, specifically promote internalization of PIN2.

## Results

### Both NAA and IAA interfere with BFA-induced intracellular cargo accumulation and endosomal aggregation

It has been shown that co-treatment of different auxins and auxin-analogues, such as IAA, NAA, 2,4-dichlorophenoxyacetic acid (2,4-d), α-(phenyl ethyl-2-one)-indole-3-acetic acid (PEO-IAA), 2-NAA, and pin static acid with BFA leads to reduced intracellular cargo (typically exemplified by PIN1 or PIN2 accumulation) in BFA bodies (Paciorek et al., 2005; Robert et al., 2010; Jasik and Elmon, 2016; Oochi et al., 2019; Paponov et al., 2019b). Since neither auxin nor BFA has been shown to inhibit transcription and translation of PINs (Paciorek et al., 2005; Vieten et al., 2005), reduced PINs and other cargoes in BFA bodies implies that auxin inhibits overall endocytosis. The visualization of a protein of interest, be it endocytosed cargo or *de novo* synthesized, secretory, or recycled protein, is facilitated by a general aggregation of the endomembrane system in response to BFA, resulting in accumulation and concentration of the protein (Satiat-Jeunemaitre and Hawes, 1994; Geldner et al., 2003; Kleine-Vehn et al., 2008a; Feraru et al., 2012; Kania et al., 2018). Already the earlier studies noted that the natural auxin, IAA, is significantly less effective than the synthetic auxin, NAA, in reducing the intracellular endocytosed cargo accumulation (Paciorek et al., 2005; Paponov et al., 2019b) and other studies using photo-convertible tag showed specifically for PIN2 a major contribution of the de novo synthesized proteins to BFA bodies formation in the presence of auxin (Jasik and Elmon, 2016). These observations do not support a specific, regulatory auxin effect on endocytosis. Therefore, we decided to resolve the contradictions by evaluating BFA as a tool for studying auxin effects. We first aimed to better characterize the NAA and IAA effects on BFA-induced endomembrane aggregation and further evaluate their effects on the intracellular accumulation of the endocytic cargo, PIN2.

Using confocal microscopy, we observed the endosomal system aggregation simultaneously with a cargo after BFA and auxin treatments. We used high BFA concentrations, such as 37.5 or 50 μM for the duration of 30–60 min to mediate the formation of bigger and more pronounced BFA bodies (Paciorek et al., 2005; Jasik and Elmon, 2016; Paponov et al., 2019b). Moreover, we used high NAA and IAA concentrations such as 10 and 20 μM to emulate the previously studied auxin-BFA effects (Paciorek et al., 2005; Robert et al., 2010; Jasik and Elmon, 2016). First we followed the aggregation of the early endosome/trans-Golgi network (EE/TGN) marked by VHA-a1-RFP (Dettmer et al., 2006) and the protein, PIN2-GFP that includes both de novo synthesized and the endocytosed cargo pools (Supplemental Figure S1B–D). To make sure that high concentrations of IAA and NAA do not affect the localization of VHA-a1 at the EE/TGN, we performed co-localization studies of VHA-a1-GFP with ARF1, a marker of Golgi and EE/TGN (Robinson et al., 2011). The study confirmed that treatment with 10 µM of IAA or NAA for a 1.5 h period did not modify VHA-a1 localization (Supplemental Figure S1A).

**Figure 1:**
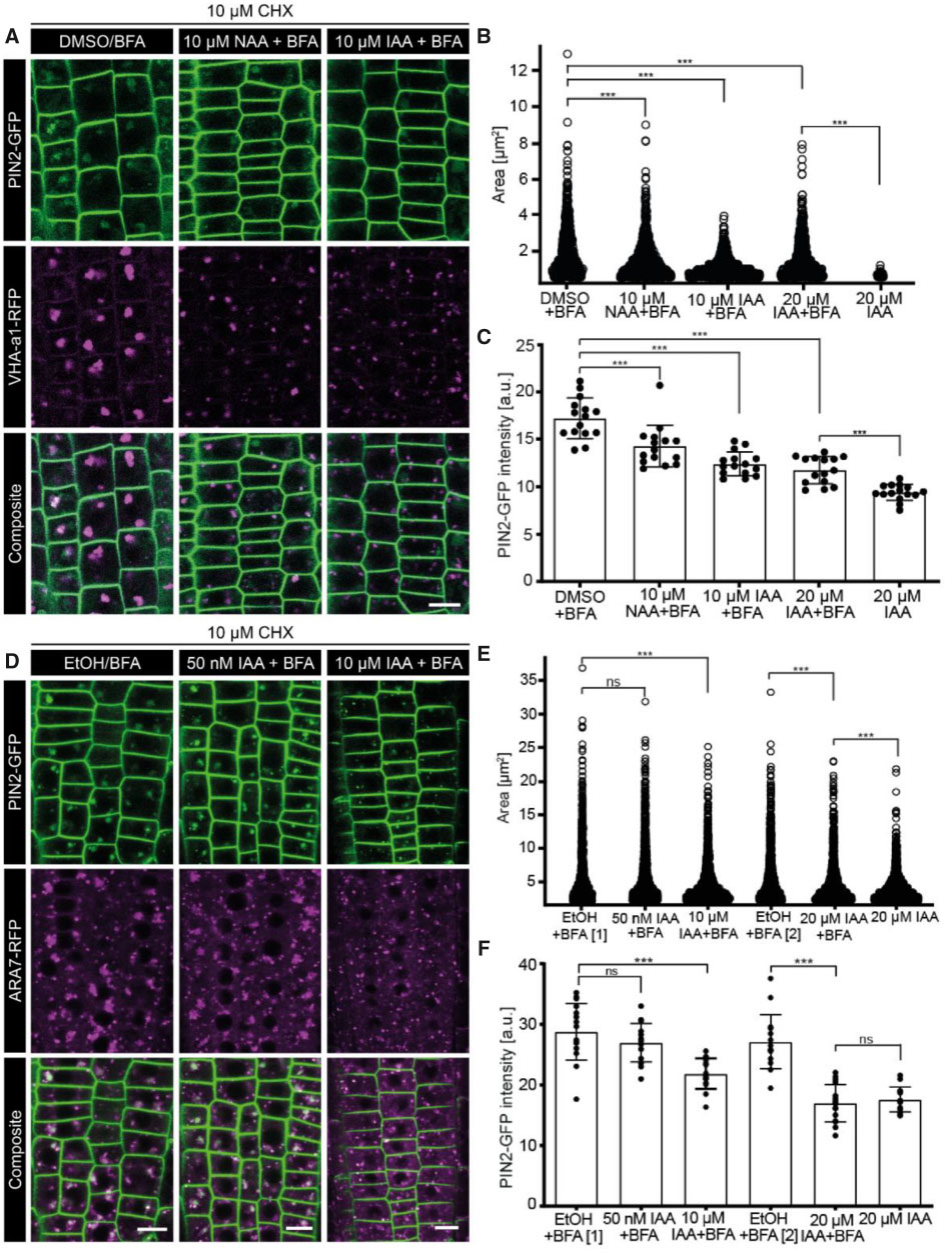
Effect of NAA and IAA on the endosomal aggregation response to BFA. A, Confocal images of the root epidermis expressing VHA-a1-RFP and PIN2-GFP. Endosomal aggregation after 30 min pre-treatment with DMSO (mock), 10 μM IAA, 20 μM IAA, or 10 μM NAA, followed by 30 min co-treatment with 50 μM BFA. 20 μM IAA treatment is the internal control. Throughout the experiment, seedlings were treated with 10 μM CHX. B, Sina plot of the EE/TGN aggregation size in μm^2^. Each point is a measurement of an aggregate. *N* = 6 roots in each condition; all the epidermal cells in the imaging plane of the root tip were measured. One-sided Mann–Whitney *U* test (653, 898, 691, 842, and 50 measurements from each condition). DMSO+BFA (mock) > 10 µM NAA+BFA, *P* = 9.646e−09***; DMSO+BFA (mock) >20 µM IAA+BFA, *P* = 6.982e−09***; DMSO+BFA (mock) >10 µM IAA+BFA, *P* < 2.2e−16***; 20 μM IAA+BFA >20 µM IAA (control), *P* < 4.59e−16***. C, Scatter dot plots of PIN2 intracellular intensity. The error bars represent mean with sd. *N* = 5 roots in each condition; 15 cells per root. One-sided *t* test (with Welch’s correction). DMSO+BFA (mock) >10 μM NAA+BFA, ***P = 0.0010; DMSO+BFA (mock) >20 μM IAA+BFA, ***P < 0.0001; DMSO+BFA (mock) >10 μM IAA+BFA, ***P < 0.0001; 20 μM IAA+BFA >20 µM IAA (control), ***P < 0.0001. D, Confocal images of root epidermal cells expressing ARA7-RFP and PIN2-GFP. Endosomal aggregation after 30 min pre-treatment with ethanol (mock), 50 nM IAA, or 10 μM IAA followed by 30 min co-treatment with 50 μM BFA. 20 µM IAA treatment is the internal control. Throughout the experiment, seedlings were treated with 10 μM CHX. E, Sina plot of the LE aggregation size in μm^2^. Each point is a measurement of an aggregate. *N* ≥ 6 roots in each condition; all the epidermal cells in the imaging plane of the root tip were measured. EtOH+BFA [1] is the corresponding mock for 50 nM and 10 µM IAA and EtOH+BFA [2] corresponds to 20 µM IAA. One-sided Mann–Whitney *U* test (1,122, 1,552, 1,397, 1,048, 1,197, and 1,229 measurements from each condition). EtOH+BFA (mock) > 10 μM IAA+BFA, ***P = 3.789e−09; EtOH+BFA (mock) >20 μM IAA+BFA, ***P = 0.000241; EtOH+BFA (Mock) >50 nM IAA+BFA, *P* = 0.2; 20 μM IAA+BFA > 20 μM IAA (control), **P = 0.001. F, Scatter dot plots of PIN2 intracellular intensity. EtOH+BFA [1] is the corresponding mock for 50 nM and 10 µM IAA and EtOH+BFA [2] corresponds to 20 µM IAA. The error bars represent mean with sd. *N* ≥ 6 roots in each condition; 15 cells per root. One-sided *t* test (with Welch’s correction). EtOH+BFA (mock) >20 μM IAA+BFA, ***P < 0.0001; EtOH+BFA (mock) >10 μM IAA+BFA, ***P < 0.0001; EtOH+BFA (Mock) >50 nM IAA+BFA, *P* = 0.11; 20 μM IAA+BFA <20 μM IAA (control), *P* = 0.26. Scale bars: 10 μm.

Under mock conditions (DMSO/EtOH + BFA), BFA bodies consisted of pronounced endosomal VHA-a1-RFP aggregations and corresponding strong accumulation of PIN2-GFP (both de novo synthesized and endocytosed) in the same structures (Supplemental Figure S1B). In combination with high concentrations of either IAA or NAA (IAA/NAA 10 μM + BFA), there was a substantial decrease in the size of the VHA-a1 aggregations. Cells contained smaller bodies of around 3 μm^2^ and the PIN2 signal in the partial aggregates was diffuse and less intense (Supplemental Figure S1B–D). We further tested the specific effects of NAA and IAA on the endocytic PIN2 pool by co-treating with the protein synthesis inhibitor cycloheximide (CHX; Obrig et al., 1971; Figure 1, A–C). The accumulation of PIN2 in the BFA bodies in the mock condition (CHX + DMSO + BFA) despite the inhibition of protein synthesis confirmed that the observed PIN2 pool is largely derived from endocytosed PM PIN2 pool and pre-existing endosomal PIN2. Consistent with previous observations, we saw that high IAA and NAA concentrations (CHX + IAA/NAA + BFA), such as 10 μM or more, interfered with the EE/TGN aggregation to form BFA bodies leading to a decreased PIN2 signal (Figure 1, A–C). We made similar observations using yet another EE/TGN marker, namely CLC2-GFP (Konopka et al., 2008), where co-treatment of BFA and NAA (10 μM) resulted in smaller CLC2-GFP-marked aggregates and concomitantly less anti-PIN2 signal in these aggregates (Supplemental Figure S1E).

These observations were further corroborated by experiments using the late endosomal (LE) marker, ARA7-RFP (Jia et al., 2013), which showed clear co-localization with PIN2-GFP already in the absence of BFA or auxin treatment (Supplemental Figure S2A). We tested the BFA-induced aggregation of LE and PIN2 cargo in the presence of both NAA and IAA with and without CHX treatment (Figure 1D–F;  Supplemental Figure S2B–D). Consistent with the EE/TGN markers, we clearly observed a concentration-dependent IAA effect on the ARA7-RFP-marked LE aggregations and concomitantly on the PIN2 cargo. At higher IAA and NAA concentrations, such as 10–20 μM, the LE aggregated only partially or not at all, and the PIN2 intensity in the BFA bodies was correspondingly low. However, at a lower IAA concentration, 50 nM, the ARA7 aggregates remained whole and aggregated, and the PIN2 intensity was high and on par with the mock condition (Figure 1D–F).

**Figure 2:**
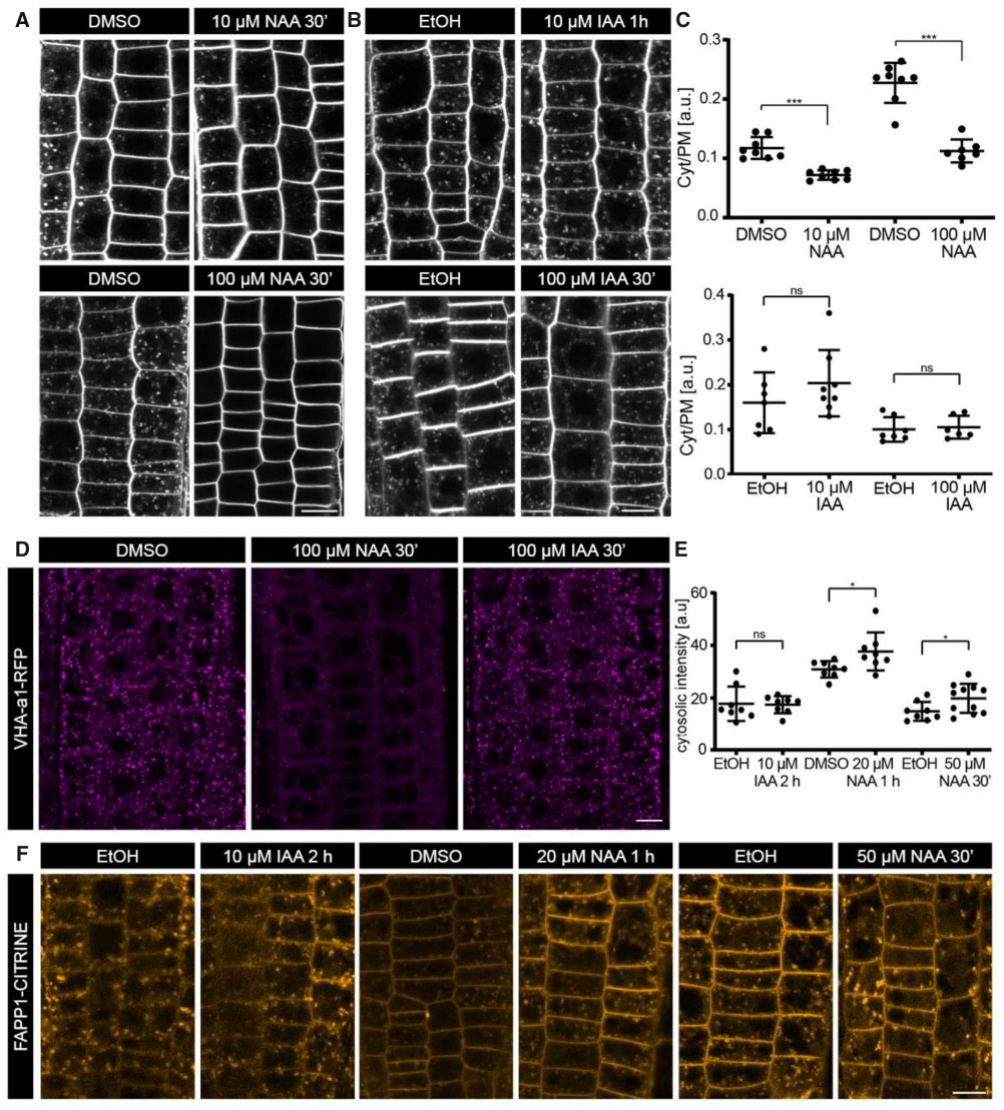
Effects of NAA and IAA on the endomembrane system. A and B, Representative confocal images of FM4-64 internalization after treatment with NAA (A) or IAA (B) (10 µM or 100 µM for 30 min). C, Scatter dot plots of the amount of endosomal FM4-64 signal measured as the ratio between the mean cytosolic intensity to the mean PM intensity. The error bars represent mean with sd. 10 µM and 100 µM NAA: *N* ≥ 7 roots per condition; at least 10 cells per root. Two-sided *t* test. DMSO (mock) versus NAA (10 µM/100 µM), *P* < 0.0001***. 10 µM IAA: *N* ≥ 6 roots; at least seven cells per root. Two-sided *t* test. EtOH (mock) versus 10 μM IAA, *P* = 0.25. 100 µM IAA: *N* = 8 roots; at least 10 cells per root. Two-sided *t* test. EtOH (mock) versus 100 μM IAA, *P* = 0.74. D, Confocal images of EE/TGN at the root epidermis expressing VHA-a1-RFP after treatment with 100 μM NAA or 100 µM IAA for 30 min. E, Scatter dot plots of the intracellular FAPP1-CITRINE intensity. The error bars represent the mean with sd. *N* = 8 roots per condition; 8 cells per root. One-sided *t* test. EtOH 2 h (mock) <10 μM IAA 2 h, *P* = 0.445; DMSO 1 h (mock) <20 μM NAA 1 h, *P = 0.0183 (with Welch’s correction); EtOH 30′ (mock) <50 μM NAA 30′, *P = 0.02. F, Confocal images of the endomembrane system marked by FAPP1-CITRINE after mock, NAA, or IAA treatment. Scale bars: 10 μm.

Finally, we observed the Golgi apparatus, whose stacks arrange at the periphery of BFA bodies around the tightly packed endosomes in their center (Satiat-Jeunemaitre et al., 1996; Naramoto et al., 2014). In the presence of 20 μM NAA or IAA (BFA + NAA/IAA), one could clearly observe disarrayed Golgi apparatus [marked by ST-YFP and anti-ARF1 (Boevink et al., 1998; Robinson et al., 2011)] around loosely dispersed EE/TGN structures [also marked by ARF1 (Robinson et al., 2011)] in less compact BFA bodies (Supplemental Figure S2E).

These observations show that high concentrations of NAA and IAA nonspecifically interfere with the aggregation of different types of endosomes and the Golgi apparatus during the formation of BFA bodies, thus leading to reduced accumulation of protein pools, be it de novo synthesized proteins or endocytosed cargo. Therefore, cargo endocytic rates are underestimated by the inability to concentrate the endocytosed cargoes in BFA bodies. Thus, the identified auxin effect on BFA-induced endosomal aggregation makes it difficult to distinguish between contributions of endomembrane aggregations and endocytic internalization of cargoes. So previous reports that auxin inhibits cargo internalization from the PM, which was based on auxin inhibiting the cargo accumulation in the BFA bodies (Paciorek et al., 2005; Robert et al., 2010), needs to be re-evaluated, preferentially using other approaches.

### NAA but not IAA inhibits the internalization of FM4-64

The inhibitory effect of auxin has so far been inferred using BFA as an indirect tool. Given the inhibitory effects of NAA and IAA on the BFA-mediated endomembrane aggregation, the interpretation of the effect of auxin on cargo endocytosis gets more complicated; hence, the question whether auxin inhibits endocytosis still remains. An alternative tool to BFA to evaluate the endocytic rate is to quantify the intracellular signal of an amphiphilic styryl dye, such as FM4-64 that stains the PM and enters cell only by membrane internalization (Jelínková et al., 2010). The internalized FM4-64-stained membranes reach the EE/TGN and over time spread over the entire endomembrane system (Rigal, 2015). Previous studies have shown that auxin decreases FM4-64 internalization into the cells, supporting the conclusion that auxin inhibits the overall endocytosis of the cell (Paciorek et al., 2005; Zwiewka et al., 2015). However, the reports were predominantly based on NAA or NAA + BFA co-treatment, and the effects of IAA have not been analyzed extensively. Therefore, we decided to evaluate the NAA and IAA concentration-dependent effects on FM 4-64 internalization.

We quantified FM4-64 internalization in the root epidermal cells at increasing NAA and IAA concentrations from 10 to 100 μM for 0.5 h (Figure 2, A–C; Supplemental Figure S3A). We detected a decrease in the intracellular FM4-64 signal at 10 μM NAA (Figure 2, A and C) and the signal became progressively weaker as the NAA concentration increased (Supplemental Figure S3A). At 100 μM, there was almost no observable intracellular FM4-64 signal (Figure 2, A and C). However, the natural auxin IAA had no effect on FM4-64 internalization even at a concentration as high as 100 μM (Figure 2, B and C). FM4-64-stained PM was still internalized and reached EE/TGN with no obvious defects/delays.

In summary, the synthetic auxin NAA inhibits FM4-64 labeling of endosomes in a concentration-dependent manner. Nonetheless, IAA, even at high concentrations, elicits no such effect.

### NAA but not IAA interferes with the structure and identity of the endomembrane system

We further investigated the observed disparity between the effects of NAA and IAA on FM4-64 internalization (Figure 2, A–C and Supplemental Figure S3A). Less intracellular FM4-64 staining after NAA could be attributed to either or a combination of: (1) inhibited endocytosis; (2) defective transport of endocytosed vesicles and endosomes along actin network; and (3) affected endomembrane system. The scenarios (2) and (3) may prove that FM4-64 internalization is not an ideal tool to study the effect of NAA on endocytosis.

We first tested the effect of NAA on endosomal movement and transport, as defective vesicular transport could result in less FM4-64 dye reaching the EE/TGN (Supplemental Video S1 and Supplemental Figure S3B). Auxin has been suggested to modify actin bundling (Rahman et al., 2007). Thus, NAA may potentially alter the transport efficiency along the cytoskeletal network as observed for the auxin transport inhibitor triiodobenzoic acid (Dhonukshe et al., 2008). Therefore, we tested the trafficking of ARA7-RFP-marked endosomes in root epidermal cells after 20 μM NAA treatment. We observed no obvious endosomal mobility defect after NAA application and the endosomal transport speed was comparable to that under mock conditions, whereas blebbistatin, a potent myosin inhibitor (Kovács et al., 2004), significantly reduced the speed (Supplemental Video S1 and Supplemental Figure S3B).

Next, we tested the effects of varying NAA and IAA concentrations on the endomembrane system itself (Figure 2, D–F; Supplemental Figure S3C). We first tested their effects on the number and distribution of EE/TGN structures in root epidermal cells, marked by VHA-a1-RFP. We observed that at very high NAA concentration (100 µM), there were far less EE/TGN structures in the cells compared to the mock condition (Figure 2D). However, at lower NAA concentrations, like 10 and 50 μM, there was no obvious decrease in the number of these structures (Supplemental Figure S3C). In contrast, IAA, even at a high concentration of 100 μM, did not affect the EE/TGN, which is also reflected by the unchanged FM4-64 signal (Figure 2, B–D). This indicates that higher concentrations of the synthetic auxin NAA have a profound impact on EE/TGN structures.

To further explore the auxin effect on the EE/TGN structures, we looked at phosphatidylinositols, the molecules that confer basic identity to the endomembrane system and that are, therefore, important for its efficient functioning (Noack and Jaillais, 2017). It was reported that NAA increases the amount of phosphatidylinositol-4,5-bisphosphate (PIns(4,5)P_2_) while decreasing the amount of phosphatidylinositol-4-monophosphate (PIns(4)P), thus altering their ratio at the PM (Tejos et al., 2014). We, therefore, tested if the observed loss of EE/TGN structures could correspond to changes in endomembrane composition (Figure 2, E and F). To this end, we applied 20 μM NAA for 1–2 h and observed the cellular PIns(4)P levels with FAPP1-Citrine biosensor (Simon et al., 2014). There was a significant increase in intracellular intensity (Figure 2, E and F), suggesting increased PIns(4)P levels at the EE/TGN. Importantly, after 50 μM NAA treatment, we observed a similar increase in intracellular intensity already after 30 min of treatment, and notably there were far less EE/TGN structures with high PIns(4)P levels (Figure 2, E and F). After 2 h of treatment, the structures completely disappeared (Supplemental Figure S3D), similar to the observations made in the VHA-a1 marker line (Figure 2D). However, IAA treatment, even after 2 h at 10 μM, had no effect on the PIns(4)P levels (Figure 2, E and F). These results indicate that NAA significantly affects the phosphatidylinositols of the EE/TGN system and, at higher concentrations, reduces the number and distribution of EE/TGN structures, which was also observed in the VHA-a1 marker line and by FM4-64 staining (Figure 2, A–D).

In summary, NAA, at high concentrations, has a profound effect on the endomembrane system, including its morphology and phosphatidylinositol composition, but IAA does not. This suggests that the observed NAA-mediated decrease in the intracellular FM4-64 staining, albeit observed already at lower concentrations, may be, at least in part, due to the affected EE/TGN structures. This effect, presumably, also contributes to the NAA effects on the BFA body formation and may provide an explanation for the previously reported auxin-mediated decrease in endocytosed cargo and intracellular FM4-64 at the EE/TGN (Paciorek et al., 2005). To unambiguously evaluate the effects of NAA on the overall endocytic rate of the cell, alternative tools, which do not involve imaging at the affected EE/TGN system, should be used. Hence, we opted to directly visualize and measure endocytosis at the PM.

### IAA and NAA do not affect the individual CME events at the PM

The synthetic auxin NAA, besides inhibiting endocytic internalization of cargoes, has also been shown to decrease the incidence of the key components of CME, like the coat protein clathrin at the cell surface (Robert et al., 2010; Grones et al., 2015). We re-evaluated these observations to determine possible direct effects of auxin on CME at the PM.

First, we re-evaluated the NAA effects on clathrin localization at the PM by visualizing CLC1-GFP (Wang et al., 2013) in root epidermal cells using confocal microscopy. After 1 h of 10 μM NAA application, we confirmed a decrease in the PM clathrin signal (Supplemental Figure S4, A and B) that potentially indicates lower density of CME events.

**Figure 3:**
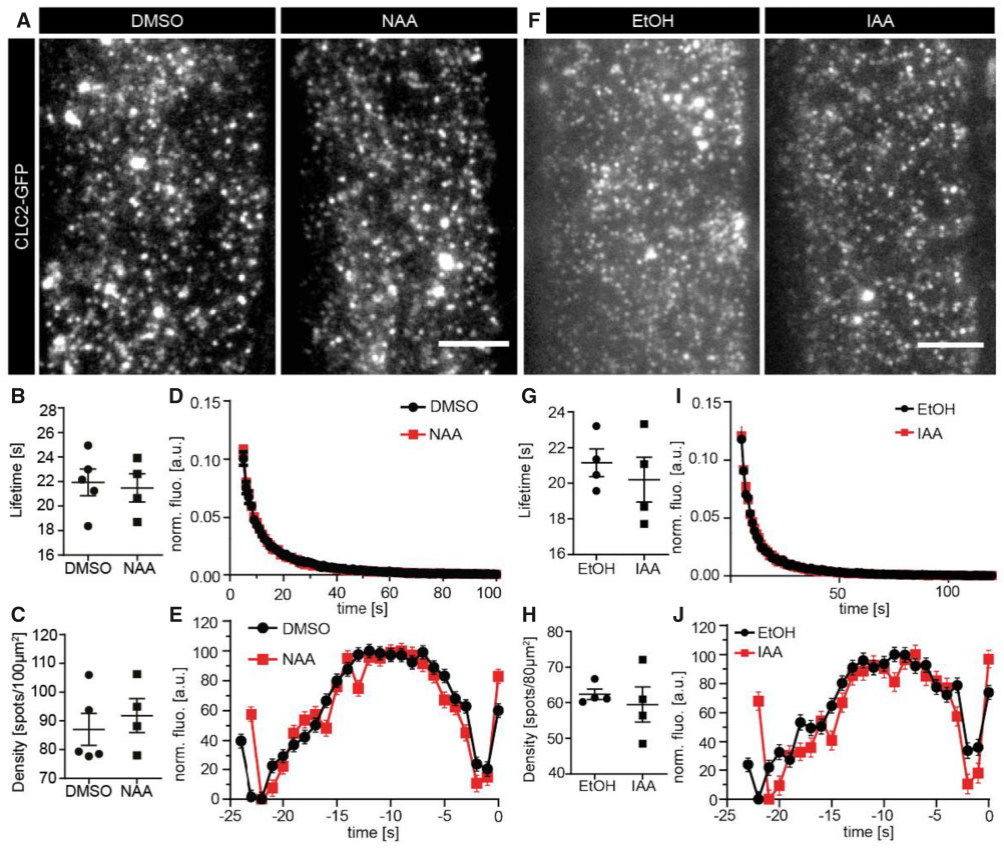
Effect of NAA and IAA on the CME machinery. A, Representative TIRF-M images of root epidermal cells expressing CLC2-GFP after treatment with DMSO (mock) or NAA (10 µM, 5–10 min). B, Mean lifetimes, (C) mean density, (D) normalized lifetime histogram, and (E) the fluorescence intensity profile of all tracks. All plots represent the mean ± se. *N*: DMSO = 5 cells from independent roots, 32,990 tracks; *N*: NAA = 4 cells from independent roots, 32,534 tracks. Two-sided *t* tests: mean lifetimes: *P* = 0.58; mean density: *P* = 0.78. F, Representative TIRF-M images of root epidermal cells expressing CLC2-GFP after treatment with DMSO (mock) or IAA (10 µM, 5–10 min). G, Mean lifetimes, (H) mean density, (I) normalized lifetime histogram, and (J) the fluorescence intensity profile of all tracks. All plots represent the mean ± se. *N*: DMSO = 4 cells from independent roots, 22,786 tracks; *N*: IAA = 4 cells from independent roots, 28,618 tracks. Two-sided *t* tests: mean lifetimes: *P* = 0.54; mean density: *P* = 0.6. Scale bars: 5 µm.

To assess a possible auxin effect on CME, we tested the effects of both NAA and IAA by directly looking at individual endocytic events at the PM of root epidermal cells using total internal reflection fluorescence (TIRF) microscopy (Johnson and Vert, 2017). We observed the endocytic foci, marked by CLC2-GFP, after short-term NAA treatment (5–10 min) at a concentration (10 µM) that showed a significant decrease in FM4-64 internalization (Figure 2, A and C) but did not cause visible endomembrane defects (Supplemental Figure S3C), thus, avoiding nonspecific effects. We saw no significant changes in the overall lifetime distribution of the endocytic foci or their density (Figure 3, A–C). Furthermore, we observed the clathrin intensity profile of all the PM foci and saw no obvious difference in the presence or absence of NAA treatment (Figure 3, D and E). The clathrin-coated pit (CCP) progressively develops as clathrin polymerizes at the pit, which can be classified into the following developmental phases: assembly, maturation, and scission. By observing the clathrin foci intensity over time, we could trace the CCP developmental profile (Loerke et al., 2009; Narasimhan et al., 2020). We analyzed the developmental profile of the foci with the average lifetime population (18–24 s) after NAA and mock treatments. The analysis did not reveal any significant differences in the duration or other characteristics of any of those phases (Supplemental Figure S4C). We further performed the same experiment with IAA (10 µM; 5 min). We observed no significant differences in CCP density, lifetime distribution, or intensity and developmental profile (Figure 3, F–J; Supplemental Figure S4D). This shows that short-term treatments of either NAA or IAA do not alter the individual clathrin endocytic foci at the PM, and by extension, the overall capacity of CME.

In summary, NAA, over the long term, decreases clathrin incidence at the PM and, consequently, could potentially alter the overall endocytic rate of the cell. However, observation of short-term effects of both NAA and IAA on a large number of individual CME events at the PM did not reveal any alterations in the incidence or dynamics of the CME machinery. Nonetheless, we cannot exclude the possibility that auxin regulates endocytosis of specific cargoes under specific conditions, for example regulatory mechanisms such as phosphorylation or ubiquitination (Mettlen et al., 2018; Zwiewka et al., 2019) instead of targeting the entire clathrin machinery at the PM.

### Both NAA and IAA promote endocytosis of PIN2 but not of other cargoes

Our analysis did not reveal any effects of NAA or IAA on the overall CME. However, we wanted to test if auxin has the potential to regulate the internalization of specific cargoes. Notably, we chose alternative tools and imaging techniques to follow the cargoes directly at the PM and not rely on their indirect observation post-endocytosis in the endosomal system (EE/TGN or LE). We first analyzed PIN2, as diverse effects of auxin on the internalization and stability of this protein are well-described (Abas et al., 2006; Baster et al., 2013). We employed the photo-convertible PIN2-Dendra (Jásik et al., 2013; Salanenka et al., 2018), which allows imaging of the pre-existing PIN2 in the red channel and the newly synthesized PIN2 pool in green after photo-conversion. Following the photo-converted PIN2 over time provides a cleaner evaluation of the rate of endocytosis.

**Figure 4:**
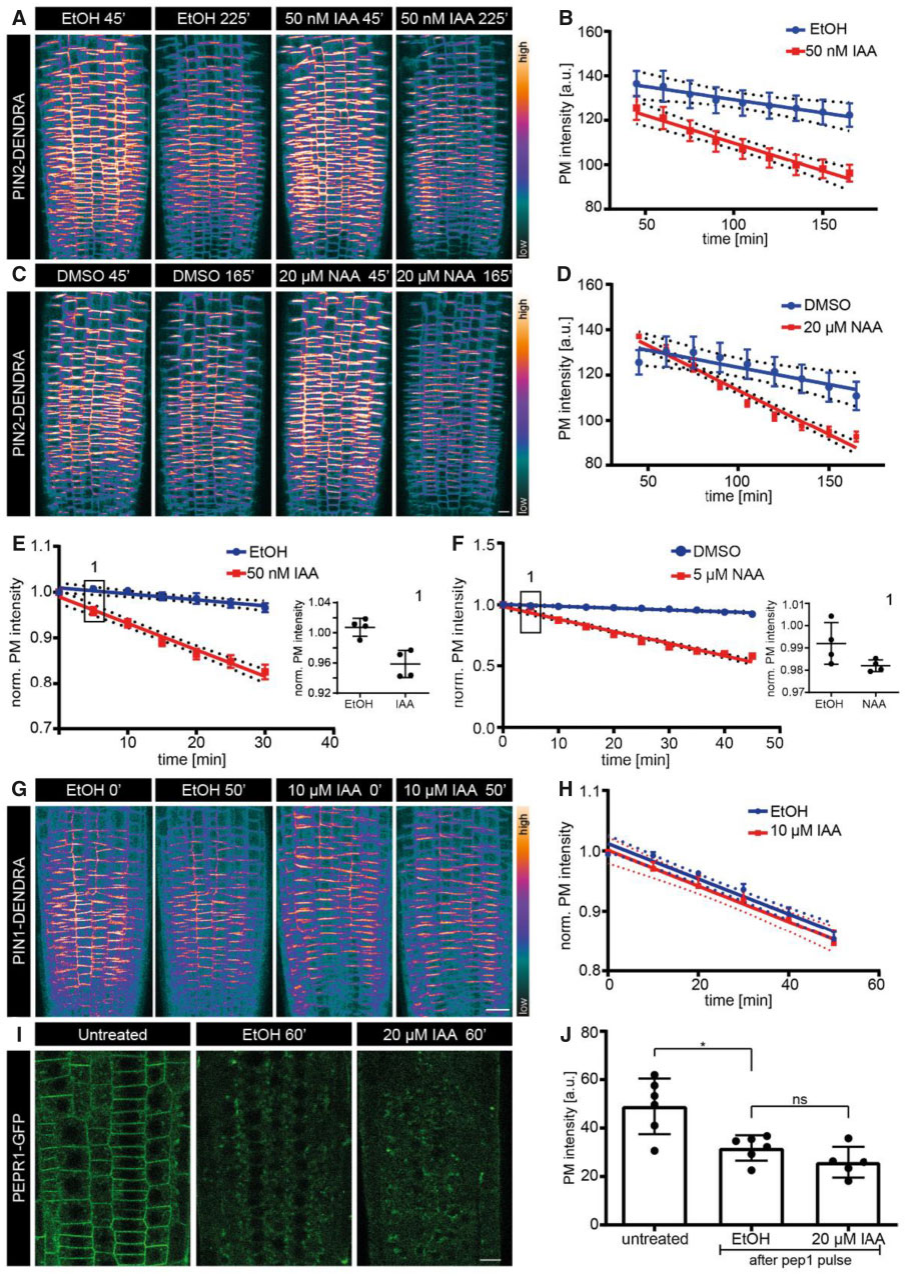
Effect of NAA and IAA on the internalization of different cargoes. A and C, Representative confocal images of the root epidermis expressing PIN2-Dendra at two isolated time-points after treatments: EtOH (mock) or 50 nM IAA (A), DMSO (mock), or 20 µM NAA (C), and their corresponding intensity measurements over-time in (B) and (D). B, Regression analysis (mock versus 50 nM IAA). *N* ≥ 3 roots per condition; two independent experiments. *χ*^2^—56.897; d*f* = 1; ***P = 4.594e−14. D, Regression analysis (mock versus 20 µM NAA). *N* = 4 roots per condition. *χ*^2^—62.216; d*f* = 1; ***P = 3.078e−15. E, Regression analysis (mock versus 50 nM IAA). *N* = 4 roots per condition. *χ*^2^—69.418; d*f* = 1; ***P = 2.2e−16. F, Regression analysis (mock versus 5 µM NAA). *N* = 4 roots per condition. *χ*^2^—75.878; *df* = 1; ***P = 2.2e−16. The scatter plot in the inset 1 (representing the first data point of graph F) shows intensity difference 5 min after IAA (E) or NAA (F) treatment. One-sided *t* test. EtOH (mock) >50 nM IAA, **P = 0.0021; DMSO (mock) >5 µM NAA, *P = 0.043. G, Representative confocal images of root epidermal cells expressing PIN1-Dendra at two isolated time-points after EtOH (mock) or 10 µM IAA treatments. H, Regression analysis (mock versus 10 µM IAA). *N* ≥ 5 roots per condition. *χ*^2^—0.65; d*f* = 1; *P* = 0.42. All the regression analyses of the PIN PM intensity were performed by fitting a linear mixed model on the intensity values measured from all the cells in the imaging plane of the epidermis. Each dot represents the mean intensity and the dotted lines depict the 95% CI. LMER—random effects for position. I, Representative confocal images of root epidermal cells expressing PEPR1-GFP; before pep1 pulse (untreated—control); and after pep1 pulse and treatments with EtOH (mock) or 20 µM IAA for 1 h. J, Scatter dot plots of PM PEPR intensity. The error bars represent the mean with sd. *N* ≥ 5 roots per condition; 10 cells per root. Two-sided Mann–Whitney *U* test. EtOH versus 20 µM IAA, *P* = 0.24. One-sided Mann–Whitney *U* test. Untreated > EtOH, *P = 0.013. Scale bars: 10 µm.

We treated the roots with varying NAA (5 μM, 10 μM, and 20 μM) and IAA (50 nM, 200 nM, and 10 μM) concentrations and followed the photo-converted PIN2 signal in the epidermal cells of the entire root tip using confocal microscopy (Figure 4, A–F; Supplemental Figure S5, A–C). With all these different concentrations of both IAA and NAA, we consistently observed a pronounced decrease in PIN2 PM signal over time compared to the mock condition (45 min to 3 h; Figure 4, A–D; Supplemental Figure S5, A–C). This suggests that auxin promotes the rate of PIN2 endocytosis from the PM, which is consistent with earlier observations (Abas et al., 2006). We further examined the lowest IAA concentration that could elicit the same response. We saw that a concentration as low as 10 nM could promote PIN2 endocytosis, albeit not as pronounced as at 50 nM or higher (Supplemental Figure S5, A and B). Next, we tested how early IAA and NAA can trigger a significant response. We used the microfluidic RootChip set-up (Fendrych et al., 2018), which allows for a controlled and fast drug application during imaging. We observed that as early as 5 min (until ≥30 min) after IAA and NAA application, promotion of PIN2 endocytosis was significant (Figure 4, E and F). These results were further confirmed by Western blots showing that the total PIN2 amount decreases substantially within 5 min of 1 μM IAA treatment (Supplemental Figure S5, D and E), meaning that PIN2 was not only endocytosed but also degraded in a rapid manner.

We then tested if auxin mediates endocytosis of only PIN2 or other efflux carriers, such as PIN1. PIN1 is localized in root endodermis and stele (Friml et al., 2002); however, we used the line PIN2::PIN1-Dendra to express PIN1 in epidermal cells like in the case of PIN2. This ensures observation of the protein-specific effects, eliminating the tissue-associated effects of auxin. We applied 10 μM IAA and followed the photo-converted PIN1 signal. Surprisingly, unlike PIN2, we observed no decrease in PIN1 PM intensity over time (Figure 4, G and H). Even at high IAA concentrations (10 μM), there was no increase or decrease over the basal constitutive endocytic rate, meaning that auxin did not influence PIN1 endocytosis.

Next, we investigated if auxin generally influences PM cargoes, or has a specific effect only on PIN2. To this end, we tested a cargo unrelated to auxin or its signaling: PEP Receptor1 (PEPR1). PEPR1 is an immune response receptor localized at the PM of the root meristem. Following binding of its signal peptide pep1, it undergoes endocytosis and subsequent degradation (Huffaker et al., 2006; Ortiz-Morea et al., 2016). After a brief pep1 pulse, we followed the PEPR1 PM signal in the presence of mock and IAA treatments (Figure 4, I and J; Supplemental Figure S5F). One hour after the pulse, we observed a substantial PEPR1 loss from the PM (untreated versus pep1 pulsed); however, there was no significant increase or decrease between the treatments (mock versus IAA; Figure 4, I and J). This implies that IAA does not alter the endocytic rate of PEPR1. Furthermore, we evaluated the long-term auxin effect on PEPR1 endocytosis by pre-treating the seedlings with NAA, IAA, or mock followed by a pep1 pulse to internalize PEPR1 in the presence of mock and auxin treatments. Once again, there was no difference in PEPR1 internalization between treatments 1 h after the pep1 pulse (Supplemental Figure S5F). This shows consistently that both endogenous and synthetic auxins (IAA and NAA) do not regulate the overall endocytosis of all cargoes, but specifically affect the PIN2 auxin transporter. This is in line with IAA not influencing the overall FM4-64 internalization (Figure 2, B and C).

In summary, by directly following a cargo unrelated to auxin, namely PEPR1, and two auxin-related cargoes, namely PIN1 and PIN2 efflux carriers, we observed that auxin promotes the endocytosis of PIN2 but does not influence other cargoes. This effect is clearly distinct from less specific effects of higher concentrations of synthetic auxins on BFA- and FM4-64 visualized endocytosis (Paciorek et al., 2005). Instead, this specific, pronounced effect on PIN2 endocytosis is elicited by both synthetic and natural auxins rapidly and at low concentration.

### Constitutive clathrin-mediated PIN2 internalization maintains apical PIN2 polarity

Inhibition of PIN endocytosis by auxin has been proposed to be a central mechanism for establishing auxin transport channels (Robert et al., 2010; Wabnik et al., 2010; Mazur et al., 2020a) and for asymmetric auxin distribution during gravitropic response (Abas et al., 2006; Baster et al., 2013). However, the physiological role of the identified auxin-mediated promotion of PIN2 internalization remains unclear.

**Figure 5:**
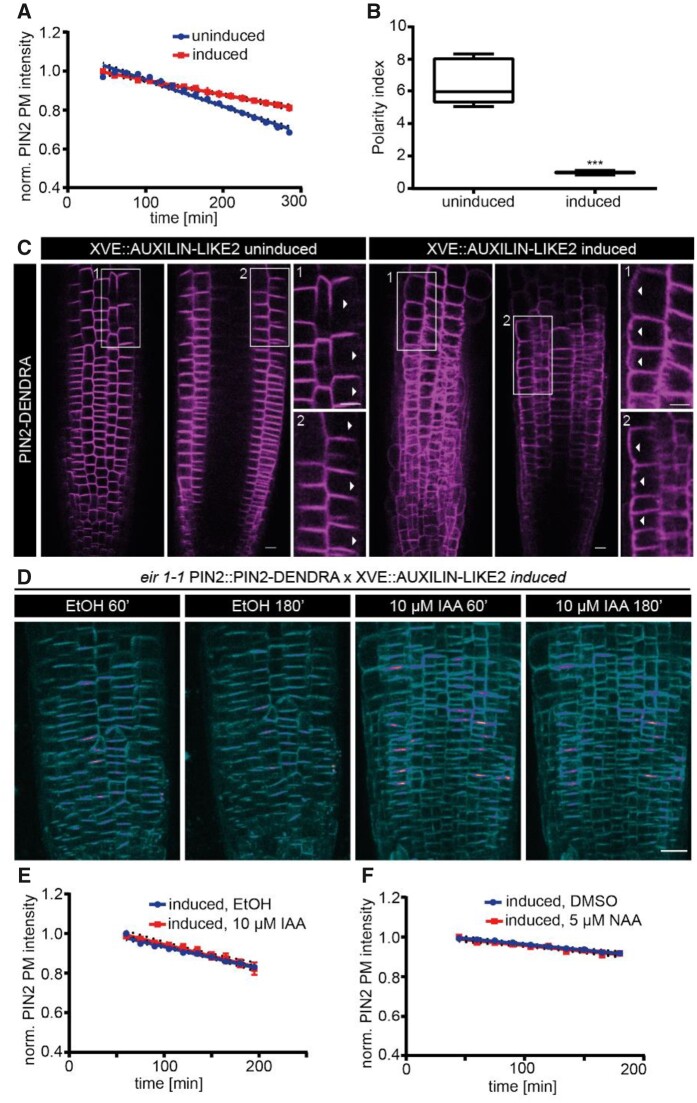
Auxin-mediated promotion of PIN2 internalization and its polarity are clathrin dependent. A, Regression analysis (uninduced versus induced). *N* = 4 roots per condition; *χ*^2^—65.82; d*f* = 1; ***P = 4.931e−16. B, PIN2 polarity index in *AXL2* uninduced versus induced conditions measured as the ratio of apical PIN2 intensity to lateral PIN2 intensity. *N* = 5 roots per condition; 10 cells per root; three independent experiments. Mann–Whitney *U* test, ***P < 0.0001. C, Representative confocal images of root epidermal cells expressing PIN2-Dendra; *AXL2* uninduced (left) and induced (right) conditions. The inset 1 in each condition shows the front of the epidermis and the inset 2 shows the middle of the epidermis. White arrowheads indicate the lateral PIN2 distribution. D, Representative confocal images of root epidermal cells expressing PIN2-Dendra at two isolated time-points after EtOH (Mock) or 10 µM IAA treatments in *AXL2*-induced condition. E, Regression analysis (mock versus 10 µM IAA). *N* ≥ 5 roots per condition; LM; *F* = 0.88; *P* = 0.34. F, Regression analysis (mock versus 5 µM NAA). *N* = 5 roots per condition; LMER—random effects for position; *χ*^2^—0.0027; *df* = 1; *P* = 0.95. The regression analysis of the PIN2 PM intensity shown in plots A and F were performed by fitting a linear mixed model on the intensity values measured from all the epidermal cells in the imaging plane of the root tip. Each dot represents the mean intensity and the dotted lines depict the 95% CI. LMER—random effects for position. Scale bar: 20 µm.

It has been shown that PINs undergo constitutive endocytosis and recycling, thus maintaining their polar distribution (Kleine-Vehn et al., 2011; Glanc et al., 2018). Previous studies have indicated that constitutive basal-rate of PIN2 internalization from the PM is clathrin-mediated (Dhonukshe et al., 2007; Kitakura et al., 2011). To this end, we monitored the basal endocytic rate of PIN2-Dendra and, furthermore, analyzed the rate after blocking CME by using the inducible over-expression line *XVE::AUXILIN-LIKE2* (Adamowski et al., 2018; Figure 5A). After inducing *AUXILIN-LIKE2 (AXL2)* over-expression for ∼24 h, we observed a significant decrease in the basal PIN2 endocytosis rate (Figure 5A). This was consistent with PIN2 constitutive endocytosis being clathrin-mediated (Dhonukshe et al., 2007; Narasimhan et al., 2020). Furthermore, when we looked at the PIN2 polarity after *AXL2* overexpression, the apical polarity was lost and there we observed an apolar PIN2 localization along the lateral sides (Figure 5, B and C). This shows that maintaining a basal rate of constitutive PIN2 endocytosis is vital for PIN2 apical polarity maintenance, as suggested before (Kleine-Vehn et al., 2011).

Then, we investigated if auxin mediates PIN2 internalization specifically through the CME pathway (Figure 5, D–F). After 24 h *AXL2* induction, we applied high IAA and NAA (10 µM) concentrations. We observed that both auxins no longer promoted PIN2 internalization (Figure 5, D–F), proving that auxin indeed promotes PIN2 internalization via CME. In parallel, we also investigated if PIN1 internalization happens through CME and further assessed if it is influenced by auxin (Supplemental Figure S6). To this end, we quantified the interaction between PIN1 and µ2 adaptin, which is necessary for cargo recognition during CME (Marcote et al., 2016), using pull-down experiments with and without NAA treatment. We observed a positive µ2-PIN1 interaction; however, there was no increase or decrease in the interaction in the presence of NAA. This proves that the constitutive endocytosis of PIN1 is clathrin-mediated, but unlike PIN2 endocytosis, it is not enhanced by auxin. Unfortunately, we failed to perform similar experiments with PIN2, presumably due to the low stability of the PIN2 protein.

Based on these results, it is conceivable that cellular auxin levels specifically regulate the basal rate of constitutive, CME-mediated PIN2 endocytosis presumably to uphold apical PIN2 polarity, and possibly contribute to polarity re-establishment.

## Discussion

In this study, we have explored the effects of both natural and synthetic auxins, IAA and NAA, on endocytosis and related trafficking processes. We show that high auxin concentrations, particularly of the synthetic auxin NAA, which interfere with uptake of the lipophilic endocytic tracer FM4-64 or with intracellular accumulation of endocytic cargoes in response to trafficking inhibitor BFA, also affect the identity and the distribution of the endomembrane system and interfere with its aggregation after BFA treatment. This renders these commonly used approaches, such as FM4-64 uptake or BFA treatment with their ineffectuality of observing the endocytosed cargo indirectly together with the endomembrane system, problematic for assessing auxin effects. By state-of-the-art imaging techniques that enable direct monitoring of the individual endocytic events or the amount of cargoes at the PM, we did not observe any general and direct effects on endocytosis but detected that both NAA and IAA rapidly promote endocytosis of PIN2 auxin transporter only. This rapid positive effect of auxin on PIN2 endocytosis may be relevant for the auxin regulation and maintenance of its polar distribution.

### Nonspecific auxin effects on the endomembrane system and BFA-sensitive trafficking

BFA treatment in Arabidopsis leads to aggregation of endosomes and other endomembrane organelles together with their endocytic, secretory, and vacuolar-targeted cargoes to form BFA bodies (Geldner et al., 2003; Paciorek et al., 2005; Kleine-Vehn et al., 2008a; Feraru et al., 2012; Kania et al., 2018). Our re-evaluation of auxin effects revealed that NAA and IAA at different concentrations interfere with BFA-induced cargo aggregations and modify the endomembrane system to a different extent. Whereas the natural auxin IAA shows mild effects, the synthetic auxin NAA is more effectual (Simon et al., 2013). The underlying cause of this difference remains unclear.

Several mutants have been described for their decreased sensitivity to the NAA inhibition of BFA-induced PIN aggregation. For example, mutations in the Callosin-like protein BIG cause auxin-insensitive, BFA-induced PIN aggregations (Paciorek et al., 2005), but the underlying mechanism remains unclear. Notably, many of these mutants have defects in the homeostasis and distribution of lipid components in the endomembrane system, including sterol biosynthesis mutants (Pan et al., 2009; Carland et al., 2010), phosphatidylserine biosynthesis (Platre et al., 2019), and aminophospholipid flippase mutants (Zhang et al., 2020). The phosphatidylserine biosynthesis mutant *pss1-3* has prominent BFA bodies, which are resistant to NAA. Additionally, *pss1-3* and ROP6^7Q^ (which has abolished phospholipid interaction) show mislocalization of ROP6 in the endosomes (Platre et al., 2019). The phospholipid translocator ALA3 flippase is localized at the EE/TGN and its mutants also have NAA-resistant prominent BFA bodies. Consistently, we found that NAA modifies the ratio of lipids, PIns (4)P, and PIns(4,5)P_2_ (Figure 2, E and F; Tejos et al., 2014). The components of the endomembrane system that are associated with the membrane lipids, such as PSST, ROP6, ALA3, and Pins(4)P, could potentially be important contributors to the BFA-induced endomembrane aggregation.

Another set of experiments showed that pH might modify the BFA effect (Supplemental Figure S7). When we incubated the roots in medium of pH 5.7, we observed PIN1-GFP in BFA bodies. The aggregates in general were smaller; additionally, the NAA effect on BFA body disaggregation was stronger. However, at pH 7.0, the BFA bodies were more pronounced and the NAA effect on disaggregation of the BFA bodies was not significant. The pH was shown to affect the membrane properties (Petelska and Figaszewski, 2000). pH and ionic homeostasis are vital for the effective functioning of the endomembrane system and also for the BFA body formation (Dejonghe et al., 2016; Sze and Chanroj, 2018). These results suggest that NAA may affect the endomembrane system through pH changes.

Jointly, these observations suggest a tight interplay between effects of auxin analogues and the endomembrane lipid composition. This could explain some effects of NAA, such as reduced number of functional endosomes, possibly also the reduced internal FM4-64 fluorescence due to a shift in spectral properties caused by a distinct membrane composition (Zal et al., 2006), and changes in the membrane potential (Dombeck et al., 2005). NAA (at 100 μM) was also found to destabilize artificial membranes, an effect that was stronger for NAA than for IAA at the same concentration (Hac-Wydro and Flasiński, 2015). This is in line with a general observation from pharmacological studies showing that synthetic compounds have often a broader activity spectrum than their natural counterparts (Feher and Schmidt, 2003).

Together, these data provide a plausible mechanistic explanation for auxin effects on the inhibition of BFA-induced endosomal aggregation, the endomembrane composition, and the observed variability between IAA and NAA effects. For additional insights, it will be of interest to dissect the mode of action and the range of effects in mutants with reported auxin-resistance in terms of the BFA effect, such as *spk1* (Lin et al., 2012), *rop6*, *ric1* (Xu, 2010), and *big/doc1/tir3* (Paciorek et al., 2005). Particularly tantalizing observations were linked to genetic manipulation of ABP1 function. Two types of conditional *abp1* knockdown lines (antisense and immunomodulation) show decreased BFA body formation. Consistently, ABP1 overexpression (whether transient in tobacco cultured cells or in stable Arabidopsis transgenic lines) leads to enhanced and NAA-resistant BFA body formation (Robert et al., 2010). Nonetheless, the conditional knockdown lines may produce off target effects (Michalko et al., 2016) and verified *abp1* knockouts show normal NAA sensitivity (Paponov et al., 2019a; Gelová et al., 2020). Thus, the possible involvement of ABP1 in auxin effect on BFA body formation remains unclear.

### Absence of direct auxin effects on clathrin-mediated endocytic events

The original observations that auxin treatment diminishes BFA-induced intracellular accumulation of endocytic cargoes as well as reduces the uptake of endocytic tracers (e.g. FM4-64) or typical CME cargoes, such as transferrin, suggested that auxin somehow targets the endocytic clathrin machinery (Paciorek et al., 2005; Robert et al., 2010). Reevaluation of this conception shows that auxin effects are much broader and include alteration of the endomembrane system. Hence, neither BFA treatment nor observing transferrin or FM4-64 dye at the EE/TGN can be used as reliable approaches to address the auxin effects on endocytosis. Hence, following the individual endocytic events at the PM directly is vital to make robust conclusions.

The notion of auxin effect on endocytosis was further supported by the observation that prolonged NAA treatment leads to a significant decrease in the clathrin density at the PM. However, the physiological relevance of this effect remains unclear. One possibility is that auxin affects the phosphatidylinositol membrane composition. For example, PIns(4,5)P2:PIns(4)P ratio at the PM increases after NAA treatment (Tejos et al., 2014) and mutants defective in PInsP metabolism, such as *pip5k1 pip5k2*, also show strongly reduced clathrin density at the PM (Ischebeck et al., 2013). Thus, membrane PInsPs are vital in maintaining the endocytic processes, and NAA, by modifying their ratios, might cause adverse effects on the CME machinery and, in extension, on the overall endocytic rate.

To address direct auxin effects on CME, we directly followed individual CME events at the PM using TIRF microscopy but did not observe any auxin effects on density or lifetime of individual endocytic foci. This suggests that auxin, despite globally influencing clathrin association with the cell surface, has no direct effects on the individual CME events, their incidence, or behavior.

### Specific, promoting auxin effect on clathrin-mediated PIN2 endocytosis

Our analyses revealed the nonspecific global effects of high auxin concentrations on endocytic processes and endomembrane functionality, and also a specific effect on endocytosis of PIN2. This is clearly a regulation distinct from the general auxin effects since the natural auxin IAA, at concentrations as low as 10 nM and rapidly within minutes, also promotes clathrin-mediated PIN2 internalization from the PM.

The potential physiological role of such auxin regulation remains unclear but may be linked to maintenance of PIN polarity, which requires constitutive PIN2 endocytosis (Dhonukshe et al., 2007; Kleine-Vehn et al., 2008c, 2011; Adamowski and Friml, 2015) and auxin-mediated PIN2 degradation (Abas et al., 2006; Baster et al., 2013). Both processes have been associated with physiological responses, such as root gravitropism, phototropism, and halotropism (Abas et al., 2006; Laxmi et al., 2008; Galvan-Ampudia et al., 2013), and their regulation occurs via many endogenous signals, such as calcium (Zhang et al., 2011) and hormones like auxin (Baster et al., 2013), gibberellic acid (Löfke et al., 2013; Salanenka et al., 2018), salicylic acid (Du et al., 2013; Tan et al., 2020), abscisic acid (Li et al., 2020), or brassinosteroids (Retzer et al., 2019). These responses typically involve not only polar cellular PIN2 distribution but also asymmetric PIN2 abundance with PIN2 stabilized on one side of the root and increased degradation on the other (Abas et al., 2006; Baster et al., 2013; Galvan-Ampudia et al., 2013). It remains to be seen how the identified auxin promotion on PIN2 internalization is connected to the regulations of gravitropic/halotropic root bending and other PIN2-mediated processes.

The signaling mechanisms underlying the auxin-mediated promotion of PIN2 endocytosis remain unclear. The less specific auxin effects on endocytosis and endomembranes do not require the canonical SCF^TIR1/AFB^ auxin pathway (Robert et al., 2010; Oochi et al., 2019), but the long-term, auxin-mediated PIN2 degradation (Baster et al., 2013) or auxin effect on PIN polarity (Sauer et al., 2006; Han et al., 2020; Mazur et al., 2020b) have been shown to require SCF^TIR1/AF^ signaling. Recently, it has been shown that SCF^TIR1/AFB^ signaling, which has been considered purely transcriptional for decades (Leyser, 2018), mediates also a nontranscriptional rapid regulation of root growth rate (Fendrych et al., 2018; Gallei et al., 2020). It is, therefore, possible that rapid PIN2 endocytosis and degradation are also mediated by the SCF^TIR1/AFB^ mechanism, but this remains to be demonstrated.

## Material and methods

###  

#### Plant material

All the plant material is from the model organism *Arabidopsis thaliana.* The marker lines used are: *pVHA-a1::VHA-a1-GFP* (Dettmer et al., 2006), *pCLC2::CLC2-GFP* (Konopka et al., 2008), *p35S::CLC1-GFP* (Wang et al., 2013), *p35S::N-ST-YFP* (Grebe et al., 2003), *pPIN2::PIN2-Dendra eir1-1* (Salanenka et al., 2018), *pPIN2::PIN2-GFP x p35S::ARA7-RFP* (Ueda et al., 2004; Xu, 2005; Zhang et al., 2016), *pUBQ10::CITRINE-1xPH(FAPP1)* (Simon et al., 2014), *pPEPR1::PEPR1-GFP pepr1 pepr2* (Ortiz-Morea et al., 2016), *XVE::AUXILIN-LIKE2 x pPIN2::PIN2-Dendra eir1-1* (Adamowski et al., 2018); *pVHA-a1::VHA-a1-RFP x pPIN2::PIN2-GFP eir1-1* was made by crossing *pVHA-a1::VHA-a1-RFP* (Dettmer et al., 2006) and *eir1-1 pPIN2::PIN2-GFP* (Xu, 2005).

#### Reagents used

IAA (indole 3-acetic acid, Duchefa Biochemie, Amsterdam, The Netherlands, I0901.0025) was dissolved in ethanol (EtOH) or DMSO (Dimethylsulfoxid) to a stock concentration of 10 mM (in DMSO), 1 mM (in DMSO), 100 μM (in EtOH), or 10 μM (in EtOH). 1-NAA (Sigma-Aldrich, St Louis, MO, N0640) dissolved in DMSO to a stock concentration of 10 mM. BFA (Brefeldin-A, Sigma-Aldrich, St Louis, MO, B7651) was dissolved in DMSO to a stock concentration of 50 mM. CHX (Sigma-Aldrich, St Louis, MO, C1988) was dissolved in DMSO to a stock concentration of 10 mM. FM4-64 (*N*-(3-triethylammoniumpropyl)-4-(6-(4-(diethylamino) phenyl) Hexatrienyl) Pyridinium Dibromide, Life Technology, T-13320) was dissolved in water to a stock concentration of 2 mM. Blebbistatin (Santa Cruz Biotechnology, San Diego, CA, sc-204253) was dissolved in DMSO to a stock concentration of 1 M. pep1 (peptide sequence: ATKVKAKQRGKEKVSSGRPGQHN (Ortiz-Morea et al., 2016), commercially synthesized by EZbiolab), was dissolved in water to a stock concentration of 200 μM. β-estradiol (Sigma-Aldrich, E8875) was dissolved in DMSO to a stock concentration of 50 mM. For Western blot analysis, the following antibodies were used: primary rabbit anti-PIN2 1:2,000 (produced and processed in lab, Abas et al., 2006), mouse anti-actin 1:5000 (Sigma-Aldrich, St Louis, MO, A0480), mouse anti-His 1:1000 (GE Healthcare, Chicago, IL) and secondary anti-rabbit IgG antibody conjugated to horseradish peroxidase (HRP) 1:10,000 (GE Healthcare, Chicago, IL, NA934). Membranes were developed using the SuperSignal Chemiluminiscence solutions (SuperSignal West Femto, Thermo Scientific, Waltham, MA). For immunolocalization the following primary and secondary antibodies were used: rabbit anti-ARF1 1:500 (Agrisera, Vännäs, Sweden, AS08325), goat anti-PIN1 1:600 (SantaCruz Technologies, San Diego, CA, sc-27163), mouse anti-GFP 1:500 (Sigma, St Louis, MO, G6539) and rabbit anti-PIN2 1:1,000 (produced and processed in lab, Abas et al., 2006), donkey anti-goat antibody coupled to Alexa Fluor 488 1:600 (Thermo Fisher Scientific, Waltham, MA, A11055), goat anti-mouse antibody coupled to Alexa Fluor 594 1:600 (Abcam, Cambridge, MA, 150116) goat anti-rabbit antibody coupled to Alexa Fluor 488 (Invitrogen, Carlsbad, CA, A11034), and sheep anti-rabbit antibody coupled to Cy3 1:600 (Sigma-Aldrich, Louis, MO, C2306).

#### Seedling growth conditions

Seeds were surface sterilized by chlorine gas and sown on 1/2 MS 0.8% agar (w/v) medium supplemented with 1% (w/v) sucrose. After stratification for 2 d in the dark at 4°C, the seedlings were grown at 21°C in a 16-h/8-h d/night cycle for 3–4 d. Seven-day-old seedlings were used for the observation of endocytic foci in roots. Five-day-old seedlings were used for PIN2 Western blot analysis. For the CLC1 PM localization experiment, 4- to 5-d-old seedlings grown under continuous light were used.

#### Pharmacological treatments

All the treatments were carried out at room temperature (RT) by diluting the drugs in liquid 1/2 MS medium containing 1% (w/v) sucrose to the working concentrations. Throughout the imaging time course, the seedlings were kept in treatment conditions, except for the FM4-64 internalization and CLC1 PM localization experiments. For the latter, imaging was done with seedlings settled flatly on a solid agar block containing the drugs dissolved to the working concentration.

BFA treatments: For Supplemental Figures S1, B–D and S2, B, seedlings were pre-treated with either DMSO, 10 μM NAA (10 mM stock), or 10 μM IAA (10 mM stock) for 30 min and then co-treated with 37.5 μM BFA (50 mM stock) and mock or the original auxin for 60 min. In Figure 1, seedlings were pre-treated with ethanol, DMSO, 50 nM (100 µM stock), 10 μM (10 mM stock), or 20 μM (10 mM stock) IAA or NAA for 30 min and then co-treated with 50 μM BFA for 30 min. 10 µM CHX (10 mM stock) treatment was present throughout the experiments. In Supplemental Figure S2E, seedlings were pre-treated with either DMSO, 20 μM NAA (10 mM stock), or 210 μM IAA (10 mM stock) for 30 min and then co-treated with 50 μM BFA (50 mM stock) and mock or the original auxin for 60 min. FM4-64 staining: seedlings were stained with 2 μM FM4-64 in liquid 1/2 MS medium. The seedlings were incubated for 2 min in the dye and washed twice before imaging. For Figure 2, A and B; Supplemental Figure S3A, seedlings were pre-treated with DMSO, ethanol, 10 μM or 100 μM IAA (10 mM stock), or varying concentrations of NAA for 30 min. Blebbistatin treatment: seedlings were pre-treated with DMSO or 500 μM Blebbistatin (1 M stock) before imaging. In Figure 4I and J, roots were pulse-treated with 200 nM (200 µM stock) pep1 for 1 min and then treated with 20 µM NAA (10 mM stock) or the corresponding mock for 60 min. As control, seedlings were not pep1 pulsed (untreated). In Supplemental Figure S5F, roots were pre-treated with 10 μM NAA, 10 μM IAA (10 mM stock), or DMSO (control) for 30 min before the pep1 pulse, followed by the same post-treatment for 60 min. As control, seedlings were incubated with DMSO/mock throughout pep1 pulse (untreated). For PIN2 Western blots, seedlings were treated with 1 µM IAA (1 mM stock) or the corresponding mock for varying durations. For β-estradiol induction, 2-d-old seedlings were transferred to plates containing 2 μM β-estradiol for 24 h. The seedlings were maintained continuously under chemical induction during subsequent imaging. Mock treatments in all experiments contained an equivalent amount of solvent in the treatment conditions.

#### Immuno-staining

For immune-staining of the roots, the InsituPro VSi robot was used as described previously (Sauer et al., 2006). Used antibodies are described in the section ‘Reagents used’.

#### Protein extraction and Western blot

Seedlings on plates were treated by spraying them with liquid 1/2 MS medium containing DMSO (mock) or 1 μM IAA. At the indicated time intervals, roots were harvested and flash frozen in liquid nitrogen. These root samples were ground using a Retsch mill for 2× 1 min at 20 Hz and the resulting root powder was re-suspended in a 1:1 (w/v) ratio of protein extraction buffer (50 mM Tris–HCl (pH 7.5), 150 mM NaCl, 1% (v/v) Triton X-100, 1× Roche complete™ Mini Protease Inhibitor Cocktail, 1× Roche PhosSTOP^TM^, 1 mM EDTA, 1 mM DTT, 10 µM MG-132, and 0.5 mM PMSF (Phenylmethylsulfonylfluorid)). The samples were incubated on ice for 30 min, with intermediate vortexing to mix root powder and extraction buffer, followed by a centrifugation step at 10,000*g* to sediment the plant debris. The cleared supernatant containing the proteins of interest was collected and the total protein content was determined using Quick Start Bradford reagent (Bio-Rad, Hercules, CA). The protein extracts were all diluted in extraction buffer to the same concentration (30 µg/25 µL) to allow equal loading of the samples. Proteins were separated by SDS–PAGE (sodium dodecyl sulfate polyacrylamide gel electrophoresis) in a 12% (v/v) acrylamide gel (Protean^®^ TGX^TM^, Bio-Rad, Hercules, CA) and were transferred to PVDF (Polyvinylidenfluorid) membranes by electroblotting (wet-transfer, Towbin transfer buffer, Bio-Rad System, Hercules, CA). The membranes were then incubated in blocking buffer (0.05% (v/v) Tween-20, 5% (w/v) milk powder or 3% (w/v) BSA, 20 mM Tris–HCl (pH 7.5), 150 mM NaCl) for at least 60 min and reacted with anti-PIN2 or anti-actin antibodies in TBS-T buffer + 3% BSA. This was followed by an anti-rabbit IgG secondary antibody conjugated to HRP incubation and chemiluminescence reaction. To allow multiple antibody detections using the same PVDF membrane, mild stripping was performed using 15 g L^−1^ glycine, 1 g L^−1^ SDS, 10 mL L^−1^ Tween-20 buffer at pH 2.2 for 2–5 min.

#### GST pull-down assay

GST and GST-PIN1CL recombinant proteins were expressed in bacteria and purified with glutathione sepharose beads, as described previously (Sancho-Andrés et al., 2016). The receptor binding domain (RBD) of Arabidopsis μ2-adaptin was expressed in bacteria as an histidine-tagged protein (His)6×-RBD-µ2-adaptin and purified with a Nickel column. Buffer exchange was performed using a PD-10 column (Amersham Pharmacia Biotech, Little Chalfont, UK) to binding buffer (100 mM Tris–HCl (pH 7.5), 5 mM EDTA, 0.1% (v/v) Triton X-100) as described previously (Sancho-Andrés et al., 2016). Purified µ2-adaptin protein was pre-incubated for 1 h at RT in the absence or presence of 10 μM NAA, 2,4-D, 2-NAA, or BA and then for 2 h at RT with 30 μL glutathione sepharose beads containing GST or GST-PIN1CL, which also had been pre-incubated with or without the respective auxin analogues. The beads were washed three times with 0.5 mL binding buffer and re-suspended in two-fold sample buffer (Laemmli, 1970). The samples were boiled at 95°C for 3 min and subjected to SDS–PAGE and Western blotting with a His-antibody. Each pull-down assay was independently performed three times and similar results were obtained.

### Microscopy

#### Confocal microscopy

To determine the PIN2 endocytic rate, photo-conversion and subsequent imaging of photo-converted PIN2-Dendra at the PM was done with a Zeiss LSM700 vertical confocal microscope using a Plan-Apochromat 20×/NA 0.8 air objective and PMT/T-PMT detectors. The whole root expressing PIN2-Dendra was photo-converted as described in Jasik et al. (2013). The growing root was tracked with the “Tip-Tracker” software as described in von Wangenheim et al. (2017). The time interval between subsequent measurements was 15 min, except for the early time point studies, where the interval was 5 min. For controlling the treatment environment during determination of the PIN2 endocytic rate (Figure 4, E and F) the roots were grown in the RootChip, as described in Fendrych et al. (2018). An alternative device, named Chip'n'Dale was designed to allow drug application during live imaging as in Figure 4J. The Chip'n'Dale device consists of a cylindrical well, a permeable polyester membrane insert and a nylon mesh in between. The cylindrical well was constructed in house. It consists of a cover glass at the bottom, four springs to allow adjustment of the depth of the well, and a notch on the edge to fit the commercial permeable polyester membrane insert (Corning). Before mounting the seedlings, a piece of nylon mesh was placed on the polyester membrane of the insert and made wet by a drop of liquid 1/2 MS medium. On the liquid medium, 4-d-old seedlings were mounted. The mounted seedlings with the insert was then flipped and clipped into the cylindrical well. Afterwards, the Chip'n'Dale with samples was mounted onto the vertical confocal microscope. The drug was injected during imaging.

Imaging of FM4-64 internalization, BFA treatment (except Figure 1A), PtdIns(4)P quantifications, and VHA-a1-RFP distribution was done with a Zeiss LSM700 inverted confocal microscope using a Plan-Apochromat 40×/NA 1.3 water objective and PMT/T-PMT detectors. Imaging of PEPR-GFP internalization was performed with a Zeiss LSM880 inverted confocal microscope using Plan-Apochromat 40×/NA 1.2 water objective and GaAsP/PMT detectors. To make the time-lapse movie of the endosomal movement and to observe the BFA effect (Figure 1A), a LSM800 inverted confocal microscope with a 40×/NA 1.3 water objective and GaAsP/PMT detectors was used. For observing the CLC1-GFP PM localization, a Zeiss LSM710 confocal microscope with a C-Apochromat 63×/NA 1.20 oil objective was used. In Supplemental Figure S7, images were taken with a Leica SP2 confocal microscopes using a 63× water objective.

#### TIRF microscopy

Roots of 7-d-old seedlings were imaged with an Olympus IX83 inverted microscope equipped with a Cell^TIRF module and Hamamatsu EM-CCD C9100-13 camera, using OLYMPUS Uapo N 100×/NA 1.49 Oil TIRF objective at 1.6× magnification. Single channel imaging was done sequentially with the mentioned time interval. Time-lapse imaging in roots was done in the epidermal cells of the transition zone in TIRF mode (Johnson et al., 2020).

### Data processing and quantification

#### Quantification of endosomal aggregation size

“Particle analysis” was performed using ImageJ to determine the sizes of the ARA7 and VHA-a1 endosome aggregates.

#### Analysis of LE movement

The time-lapse movie of LE movement was processed using ImageJ. The frames were stabilized and then subjected to temporal color-coding (Magenta Hot).

#### Quantification of endocytic foci at the PM

Time-lapse data sets of CLC2-GFP were processed as using the unbiased automated single channel endocytosis analysis in Matlab, as described in Narasimhan et al. (2020). The detections were made using the values of the experimental setup. The developmental profile of the endocytic foci marked by CLC2-GFP was processed as described in Narasimhan et al. (2020). Post processing of the data and the subsequent plots were made in GraphPad Prism6 (GraphPad Software, La Jolla, CA).

#### PIN2 endocytic rate test

The time series were processed using ImageJ. Maximum intensity Z-projection of the epidermal PIN2 signal was made and a ROI (region of interest) covering the majority of the epidermal cells of the root tip was drawn. Within this ROI, the mean intensity of photo-converted PIN2-Dendra was measured over time using the multi-measure option.

#### Other intensity quantifications

PM and/or cytosolic signal intensity measurements for analyzing PEPR-GFP localization, FM4-64 staining, PIN2 polarity, and PIN2 visualization in the aggregates were done by drawing free-hand lines at the PM and polygons internally in the individual cells, followed by measuring the mean intensity values in these regions using ImageJ. The number of cells with strong PM CLC1-GFP signal was visually evaluated and counted.

### Statistical analysis

A logistic regression was performed to compare the presence of CLC1-GFP at the PM in root cells of roots treated with DMSO versus roots treated with 10 μM NAA. A random effect was added to the model for the experiments with multiple repeats to take into account the correlation between measurements done at the same time. The analysis was performed with the glimmix procedure from SAS (Version 9.4 of the SAS System for windows 7 64bit. Copyright 2002–2012 SAS Institute Inc. Cary, NC, USA [www.sas.com]). Maximum likelihood estimation was done with the default estimation method. A Wald-type test was performed to estimate the effect of the treatment on the localization of CLC1-GFP at the PM.

Statistical analyses for differences in PIN2 internalization rate between treatments were carried out using R (version 1.1.383). A linear mixed effects regression (LMER) was used to test for the effect on the PIN2 internalization rate. We modeled PIN2 PM intensity values as a function of two predictors: time and treatment and their interaction, and we included a random intercept for each root, which is common for longitudinal studies (Bolker et al., 2009). We assessed the model’s significance comparing it to a null (mean) model and the significance of the interaction comparing to a model without interaction using likelihood ratio tests. The modeling package lme4 was used (Bates et al., 2014). The model assumptions were checked by (1) testing for equal variance of the residuals, (2) testing for normality of the residuals, and (3) testing the normality of the random effects. For statistical analysis of the immunolocalization of PIN2 in BFA bodies, a logistic regression was performed to compare the presence of BFA bodies in root cells of untreated roots versus treated roots or wild-type versus mutant. A random effect was added to the model for the experiments with multiple repeats to consider the correlation between measurements done at the same time. The analysis was performed with the glimmix procedure from SAS (Version 9.4 of the SAS System for windows 7 64bit. Copyright 2002–2012 SAS Institute Inc. Cary, NC, USA [www.sas.com]). Maximum-likelihood estimation was done with the default estimation method. A Wald-type test was performed to estimate the treatment/genotype effect on the presence of BFA bodies in the root cells.

The endosomal aggregation size was analyzed in R. The statistical tests for all the other experiments were made in GraphPad Prism 6. Significance is defined by *P* < 0.05. The number of samples, the repetitions, and the type of statistical tests are described in the respective figure legends.

### Accession numbers

Sequence data from this article can be found in the GenBank/EMBL data libraries under accession numbers ARA7—AT4G19640, CLC1—AT2G20760, CLC2—AT2G40060, ADAPTIN µ2—AT5G46630, VHA-a1—At2g28520, PIN1—AT1G73590, PIN2—AT5G57090, AUXILIN-LIKE2—AT4G12770, PEPR1—AT1G73080, and PEPR2—AT1G17750.

## Supplemental Data

The following materials are available in the online version of this article.

**Supplemental Figure S1.** Effect of NAA and IAA on EE/TGN system and its BFA-induced aggregation.

**Supplemental Figure S2.** Effect of NAA and IAA on BFA-induced aggregation of LE and Golgi bodies.

**Supplemental Figure S3.** Effects of NAA and IAA on the endomembrane system.

**Supplemental Figure S4.** Effects of NAA and IAA on PM clathrin.

**Supplemental Figure S5.** Effect of NAA and IAA on internalization of different cargoes.

**Supplemental Figure S6.** Effect of auxin analogues on binding of µ2-adaptin to the cytosolic loop of PIN1.

**Supplemental Figure S7.** Effect of pH on BFA body formation.

**Supplemental Movie S1**. Effect of NAA on endosomal movement.

## Supplementary Material

kiab134_Supplementary_Data

## Notes

M.N., M.A., and J.F. conceived the idea. M.N. designed and performed the experiments, analyzed the data and wrote the paper. M.G. performed BFA experiments and co-wrote the paper. S.T., R.W., and L.R. co-performed BFA and FM4-64 based experiments. A.J. and E.H. performed experiments on CME machinery. H.H made the trafficking movie. L.L. assisted in Chip’n’Dale and Rootchip experiments. I.V. and J.S. performed the protein work. F.A., S.V., and J.F. supervised the work.

The author responsible for distribution of materials integral to the findings presented in this article in accordance with the policy described in the Instructions for Authors (https://academic.oup.com/plphys/pages/general-instructions) is: Madhumitha Narasimhan (jiri.friml@ist.ac.at).
