# Corrigendum to: Systematic analysis of specific and nonspecific auxin effects on endocytosis and trafficking

**DOI:** 10.1093/plphys/kiab380

**Published:** 2021-10-04

**Authors:** Madhumitha Narasimhan, Michelle Gallei, Shutang Tan, Alexander Johnson, Inge Verstraeten, Lanxin Li, Lesia Rodriguez, Huibin Han, Ellie Himschoot, Ren Wang, Steffen Vanneste, Judit Sánchez-Simarro, Fernando Aniento, Maciek Adamowski, Jiří Friml

*Plant Physiology*, Volume 186, Issue 2, June 2021, Pages 1122–1142, https://doi.org/10.1093/plphys/kiab134

An additional affiliation for Stefan Vanneste was omitted from the above paper. Dr. Vanneste’s affiliations should be as follows:

1. Department of Plant Biotechnology and Bioinformatics, Ghent University, Ghent, Belgium

2. VIB Center for Plant Systems Biology, Ghent, Belgium

