# Supplementary material for: Systematic analysis of specific and nonspecific auxin effects on endocytosis and trafficking
Source: Plant Physiol. 2021 Mar 18;186(2):1122–42. doi: 10.1093/plphys/kiab134 (PMC8195513; doi:10.1093/plphys/kiab134)
Supplement: kiab134_Supplementary_Data [file kiab134_supplementary_data.zip › pp.00723.2020-s01.pdf]

Supplemental Figure S1: **Effect of NAA and IAA on EE/TGN system and its BFA-induced aggregation**

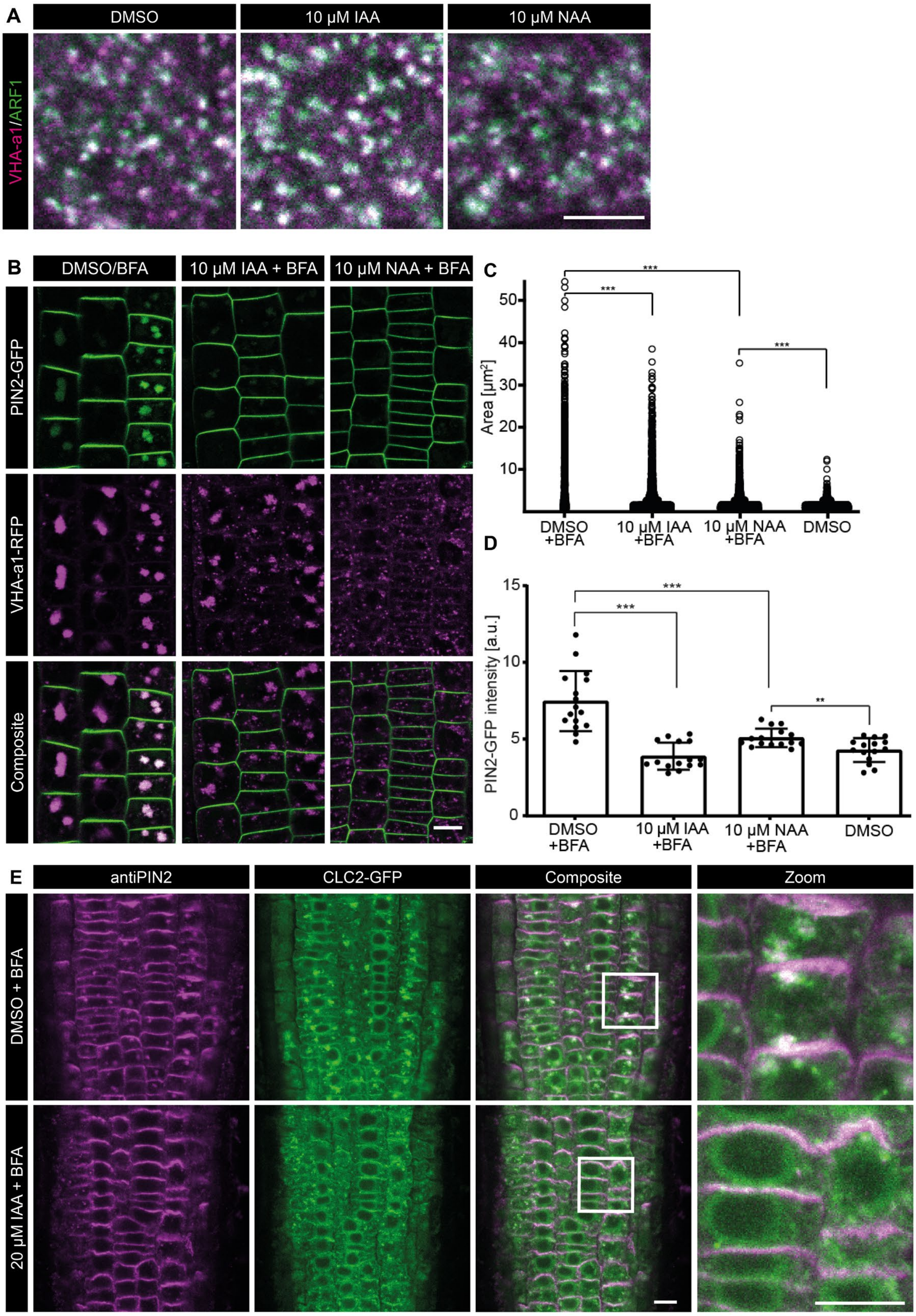

A) Representative confocal images of the root epidermis expressing VHA-a1-GFP, immunolabeled with anti-GFP to visualize VHA-a1 and anti-ARF1 antibody. The zoomed-in images show co-localization of ARF1 and VHA-a1 in

the EE/TGN system in DMSO (mock) or auxin-treated conditions (10 µM IAA and 10 µM NAA; 1.5 h). N=4 roots per condition. **B)** Representative confocal images of the root epidermis expressing VHA-a1-RFP and PIN2-GFP. EE/TGN aggregation after 30 min pre-treatment with DMSO (mock), 10 µM IAA or 10 µM NAA, followed by 60 min co-treatment with 37.5 µM BFA. **C)** Sina plot representing the EE/TGN aggregation size in µm^2. Each point is a measurement of an aggregate. N=5 roots in each condition; all the epidermal cells in the imaging plane of the root tip were measured. One-sided Mann-Whitney U test (858, 2064, 2220 and 2550 measurements from each condition). DMSO+BFA (mock) > 10 µM IAA+BFA, p<2.2e-16***; DMSO+BFA (Mock) > 10 µM NAA+BFA, p<2.2e-16***; 10 µM NAA+BFA > DMSO (control), p<2.2e-16*** . **D)** Scatter dot plots representing the PIN2 intracellular intensity. The error bars represent mean with SD. N=5 roots in each condition; 15 cells per root. One-sided t test (with Welch's correction). DMSO+BFA (mock) > 10 µM IAA+BFA, p<0.0001***; DMSO+BFA (mock) > 10 µM NAA, p<0.0001***; 10 µM NAA+BFA > DMSO (control), p=0.002**. **E)** Representative confocal images of root epidermal cell expressing CLC2-GFP immunolabeled with anti-PIN2 antibody. The roots were pre-treated with DMSO (mock) or 20 µM IAA for 30 min, followed by a co-treatment with 50 µM BFA for 30 min, and then immunolabeled. N=8 roots per condition. Scale bars: A) 5 µm B, E) 10 µm.

Supplemental Figure S2: **Effect of NAA and IAA on BFA-induced aggregation of LE and Golgi bodies**

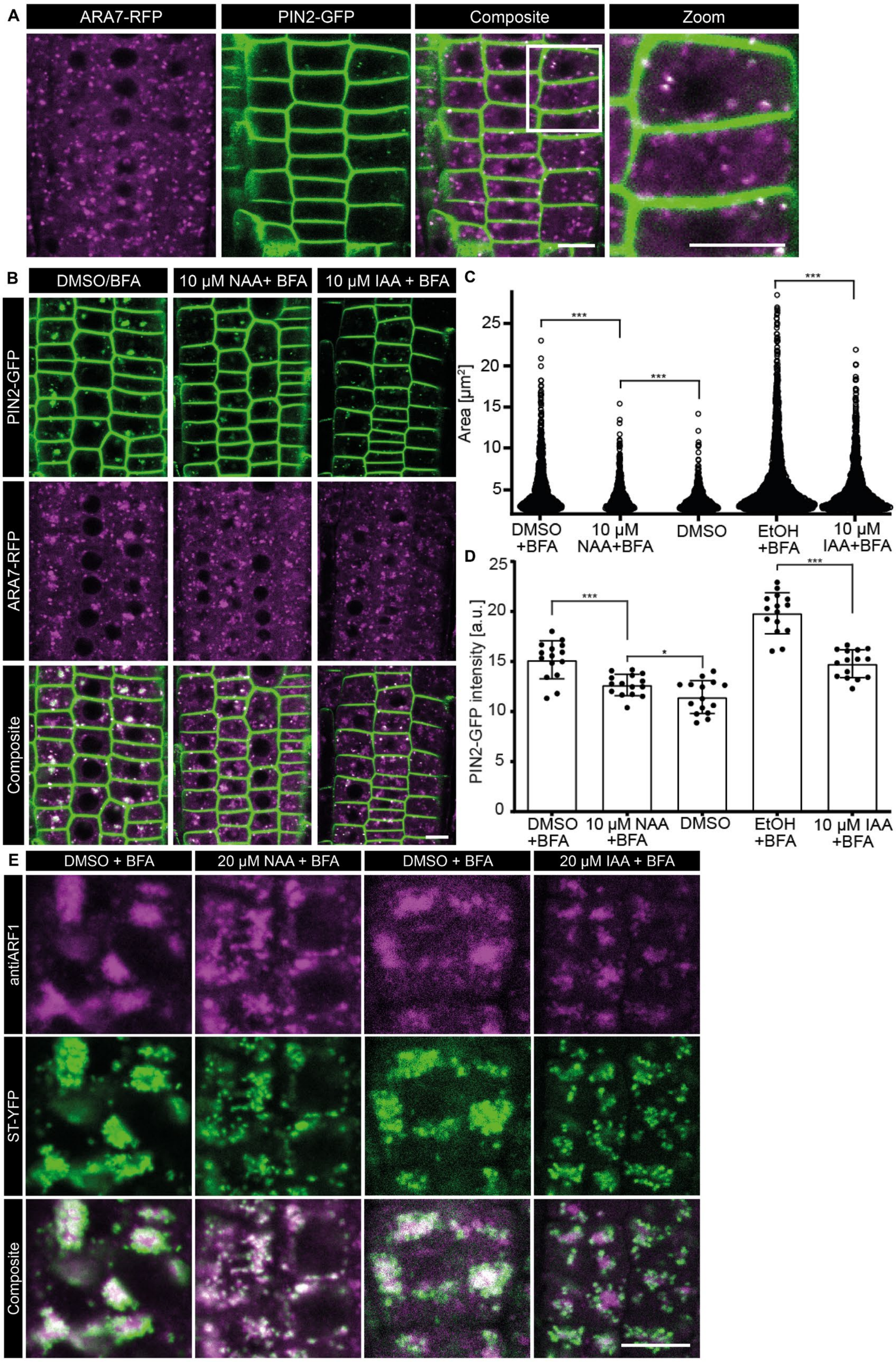

A and B) Representative confocal images of root epidermal cells expressing ARA7-RFP and PIN2-GFP. **A)** The image shows the co-localisation of PIN2-GFP with ARA7-RFP under no external treatment. **B)** LE aggregation after 30 min pre-treatment with DMSO (mock), 10 µM IAA or 10 µM NAA, followed by 30 min co-treatment with

50 µM BFA. **C)** Sina plot representing the LE aggregation size in µm2. Each point is a measurement of an aggregate. N=8 roots in each condition; all the epidermal cells in the imaging plane of the root tip were measured. One-sided Mann-Whitney U test (869, 603, 578, 1585 and 1403 measurements from each condition). DMSO+BFA (mock) > 10 µM NAA+BFA, p-value = 7.997e-06***; EtOH+BFA (Mock) > 10 µM IAA+BFA, p-value = 1.606e-10 ***; 10 µM NAA+BFA > DMSO (control), p-value = 0.0001922 ***. **D)** Scatter dot plots representing the PIN2 intracellular intensity. The error bars represent mean with SD. N=8 roots in each condition; 15 cells per root. One-sided t test (with Welch's correction). DMSO+BFA (mock) > 10 µM NAA, p<0.0001***; EtOH+BFA (mock) > 10 µM IAA +BFA, p<0.0001***; 10 µM NAA+BFA > DMSO (control), p=0.01*. **E)** Representative confocal images of the root epidermal cells expressing ST-YFP, immunolabeled with anti-ARF1 antibody. The roots were pre-treated with DMSO (mock) or 20 µM IAA or 20 µM NAA for 30 min, followed by a co-treatment with 50 µM BFA for 30 min, and then immunolabeled. N=8 roots per condition. Scale bars: A,B,E) 10 µm.

Supplemental Figure S3: **Effects of NAA and IAA on the endomembrane system**

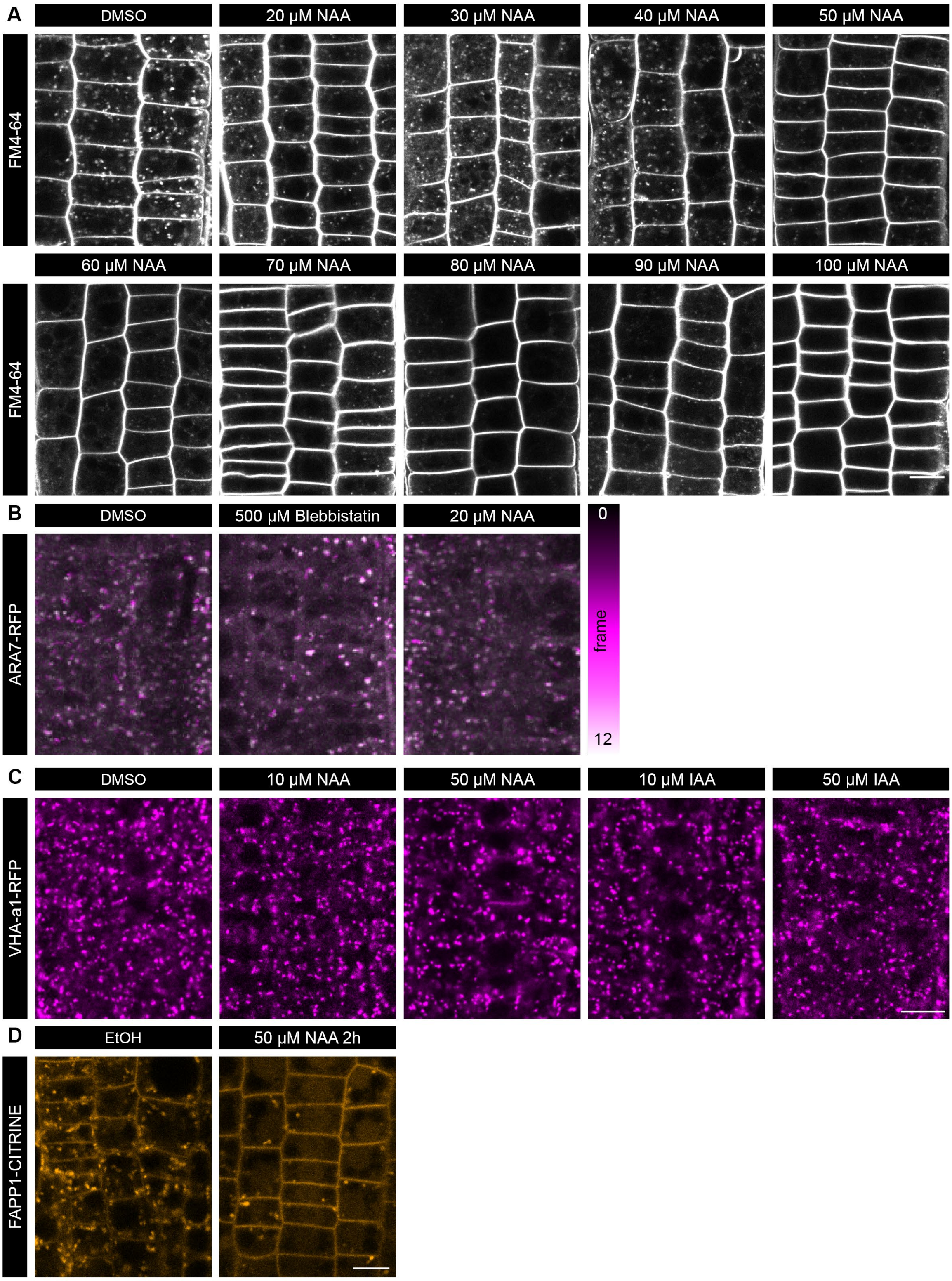

A) Representative confocal images of epidermal cells of the root with internalized FM4-64 dye marking the endomembrane after 30 min pre-treatments with DMSO (mock) or NAA: 10 to 100 µM. N=10 roots for each condition. **B)** Temporal color code of the time-lapse movie (Movie S1) of the LE movements in the roots expressing ARA7-RFP, after DMSO (mock), 500 µM blebbistatin or 20 µM NAA for 60 min. The frames are color-

coded as the color-scale bar indicates. The whiter the endosomes are, the lesser the movement. **C)** Representative confocal images of root epidermal cells expressing VHAa1-RFP after treatments with DMSO (mock), NAA or IAA for 30 min. N≥7 roots per condition. **D)** Confocal images of the endomembrane system marked by FAPP1-CITRINE after mock (EtOH) or 50 µM NAA treatment for 2 h. N≥8 roots per condition. Scale bars: 10 µm.

Supplemental Figure S4: **Effects of NAA and IAA on PM clathrin**

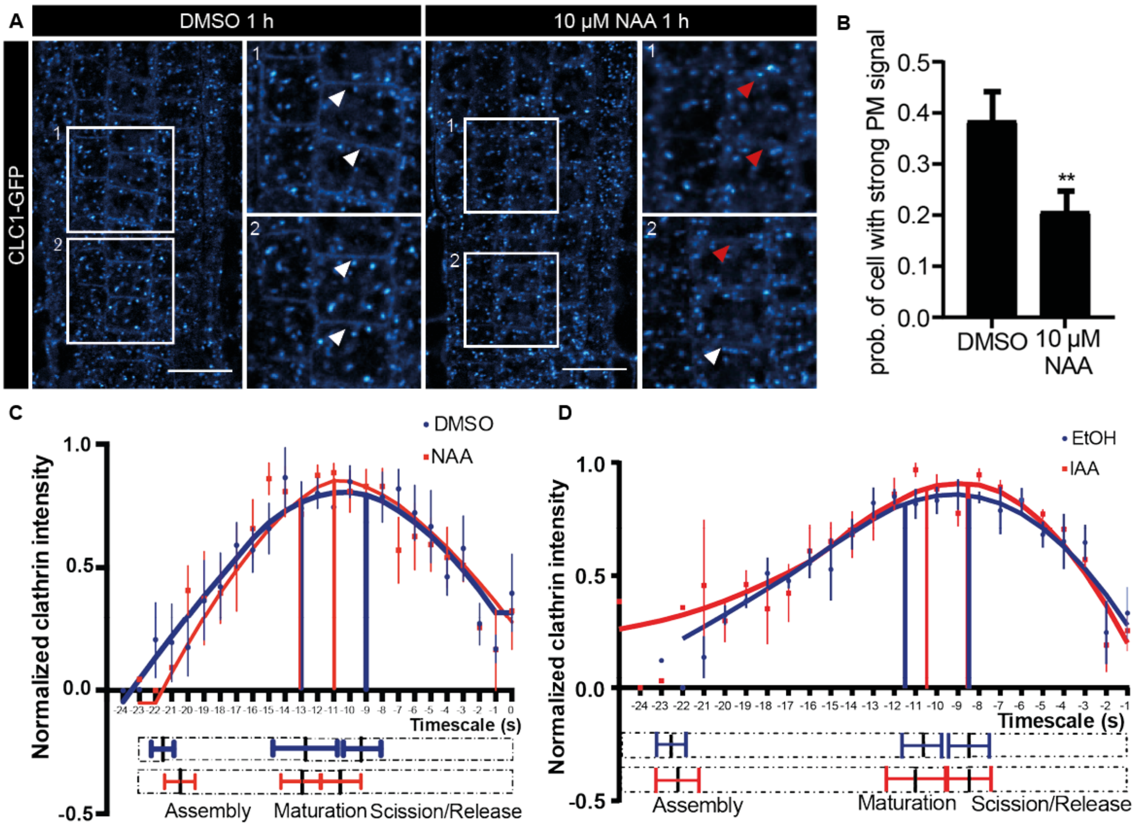

A) Representative confocal images of the root epidermal cells expressing CLC1-GFP. Red arrows indicate weak/loss of PM CLC1 signal and the white arrows indicate a strong signal. **B)** Bar graph showing the frequency of cells with strong CLC1 PM signal (10 µM NAA, 1 h). Error bars indicate 95% CI. N=4-12 roots; 6-33 cells per root; 4 independent experiments. Two-sided t test. p<0.01*. **C and D)** Smoothened intensity profile of clathrin tracks of the mean lifetime (18-24 s) after mock and auxin (10 µM NAA or IAA; 5-10 min). Each dot represents the mean intensity with SEM. CCP developmental profile classified into 'Assembly', 'Maturation' and 'Scission/Release' phases (bottom). The extrapolation lines mark the different CCP development phases. The dotted bars represent the whole time course of CCP development; the solid lines with error bars mark the mean ± SD of the transition point between phases. **C)** N: DMSO=4 cells from independent roots, 397 tracks; NAA=4 cells from independent roots, 783 tracks. Two-sided t tests. Assembly p=0.69; Maturation p=0.48. **D)** N: EtOH=4 cells from independent roots, 247 tracks; IAA=4 cells from independent roots, 431 tracks. Two-sided t tests. Assembly p=0.58; Maturation p=0.73. Scale bar: 20 µm.

Supplemental Figure S5: **Effect of NAA and IAA on internalization of different cargoes**

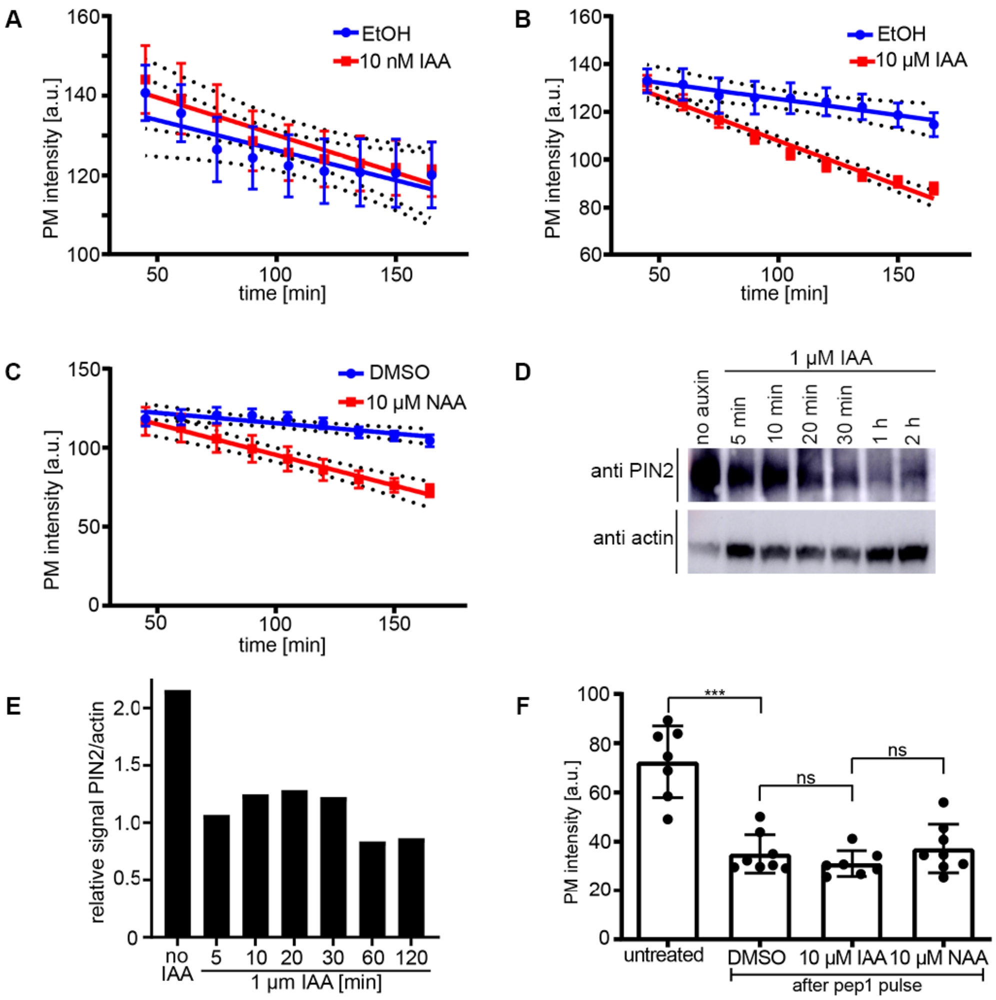

A-C) All the regression analysis of the photo-converted PIN2 PM intensity were performed by fitting a linear mixed model on the intensity values measured from all the epidermal cells in the imaging plane of the root tip. The error bars represent the mean with SE. Each dot represents the mean intensity and the dotted lines depict the 95% CI. LMER - random effects for position. **A)** Regression analysis (mock vs. 10 nM IAA): N=5 roots per condition; $\chi2$- 15.70; p=7.187e-05 ***. **B)** Regression analysis (10 µM IAA): N=5 roots per condition; $\chi2$- 96.703; p=2.2e-16 ***. **C)** Regression analysis (mock vs. 10 µM NAA): N=3 roots per condition; $\chi2$- 50.17; p=1.41e-12 ***. **D)** Western blot showing the total amount of PIN2 without auxin treatment (control) and after different incubation times with 1 µM IAA. Anti-actin was used as a loading control. **E)** Bar graph representing the relative amount of PIN2 intensity to intensity of the anti actin loading control. **F)** The scatter dot plot of the mean PM PEPR intensity with no pep1 pulse (untreated – control) or after pep1 pulse in the presence DMSO (mock) or auxin treatment (30 min pretreatment followed by pep1 pulse and then 1 h treatment with DMSO, 10 µM IAA or 10 µM NAA). The error bars represent the mean with SD. $N \geq 7$ seedlings per condition; 20 cells per root. Two-sided Mann-Whitney U test. DMSO (mock) vs. 10 µM IAA, p = 0.27; DMSO (mock) vs. 10 µM NAA, p=0.56; One-sided Mann-Whitney U test. Untreated > DMSO (mock), p=0.003**.

Supplemental Figure S6: **Effect of auxin analogues on binding of µ2-adaptin to the cytosolic loop of PIN1**

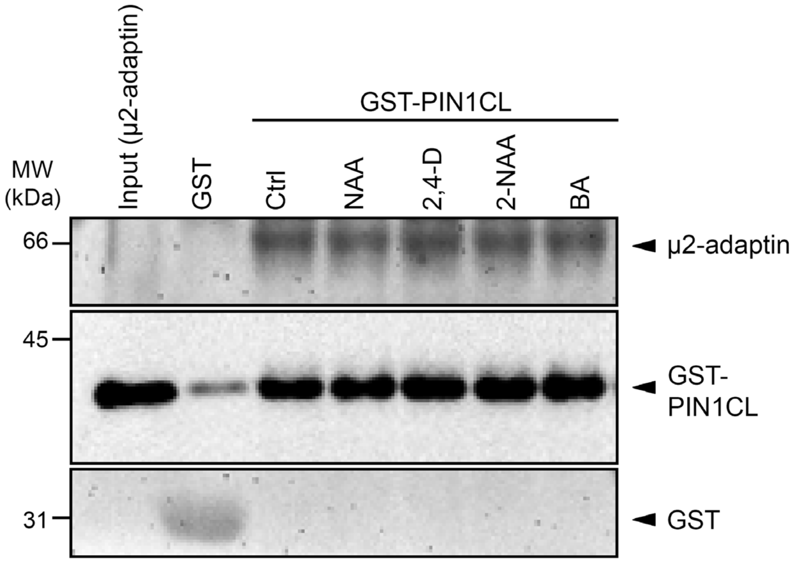

GST Pull-down assays showing the binding of the cytosolic loop of PIN1 fused with GST (GST-PIN1CL) and the receptor binding domain (RBD) of *Arabidopsis* µ2-adaptin, with an N-terminal $(His)_6$-tag used for purification and detection, in the absence (control) or presence of BA (mock) or auxin analogs (10 µM of NAA, 2,4-D, 2-NAA). GST was used as a control. Pull-downs were analyzed by Western blotting with an anti-His antibody, which detected His-tagged µ2-adaptin (upper panel), and Ponceau staining (medium and bottom panels) showing the loaded amount of GST and GST-PIN1CL in the pull-down assays. The input lane contains 5% of the amount of the µ2-adaptin used in the pull-down assay. Arrowheads point at the expected positions of the RBD of µ2-adaptin, GST and GST-PIN1CL.

Supplemental Figure S7: **Effect of pH on BFA body formation**

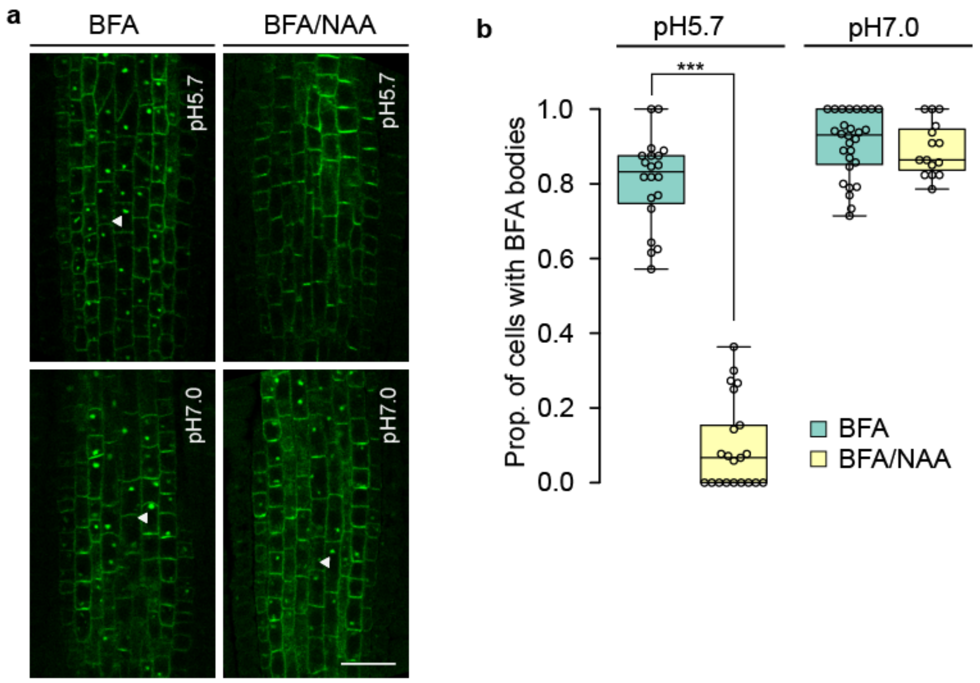

A) Representative confocal images of root endodermal and stele cells with PIN1 immunolocalization after 1 h treatment with with 25 µM BFA or co-treatment with 25 µM BFA and 10 µM NAA in liquid medium set at pH 5.7 and pH 7.0. White arrows indicate PIN1 in BFA bodies. Scale bar: 20 µm. **B)** Quantification of the probability of BFA bodies in the root cells. Center-lines show the medians; box limits indicate the 25th and 75th percentiles; whiskers extend 1.5 times the interquartile range from the 25th and 75th percentiles. pH 5.7: N=21 roots; 28 cells; $p \leq 0.0005$***. pH 7.0: N=29 roots; 28 cells; p=n.s. 5 independent experiments.

Supplemental Movie S1: **Effect of NAA on endosomal movement**

Representative time-lapse movie of LE movements in roots expressing ARA7-RFP. Roots were treated with the following drugs for 1 h and the LEs were observed by confocal microscopy in 4s intervals. Positive control: 500 µM blebbistatin; Mock: DMSO; 20 µM NAA; N=6 roots per condition; 2 independent experiments.
